# Cost-effectiveness of finerenone in chronic kidney disease associated with type 2 diabetes in The Netherlands

**DOI:** 10.1186/s12933-023-02053-6

**Published:** 2023-11-28

**Authors:** Sara W. Quist, Alexander V. van Schoonhoven, Stephan J. L. Bakker, Michał Pochopień, Maarten J. Postma, Jeanni M. T. van Loon, Jeroen H. J. Paulissen

**Affiliations:** 1 Department of Health Sciences, University of Groningen, Groningen, The Netherlands; 2 Asc Academics, Groningen, The Netherlands; 3 Division of Nephrology, Department of Internal Medicine, University Hospital Groningen, Groningen, The Netherlands; 4 Assignity, Kraków, Poland; 5 Department of Economics, Econometrics and Finance, University of Groningen, Groningen, The Netherlands; 6 Value-XS, Houten, The Netherlands

**Keywords:** Cost-effectiveness-analysis, Chronic kidney disease, Type 2 diabetes, Costs, QALYs, Finerenone

## Abstract

**Background:**

In the Netherlands, more than one million patients have type 2 diabetes (T2D), and approximately 36% of these patients have chronic kidney disease (CKD). Yearly medical costs related to T2D and CKD account for approximately €1.3 billion and €805 million, respectively. The FIDELIO-DKD trial showed that the addition of finerenone to the standard of care (SoC) lowers the risk of CKD progression and cardiovascular (CV) events in patients with CKD stages 2–4 associated with T2D. This study investigates the cost-effectiveness of adding finerenone to the SoC of patients with advanced CKD and T2D compared to SoC monotherapy.

**Methods:**

The validated FINE-CKD model is a Markov cohort model which simulates the disease pathway of patients over a lifetime time horizon. The model was adapted to reflect the Dutch societal perspective. The model estimated the incremental costs, utilities, and incremental cost-effectiveness ratio (ICER). Sensitivity and scenario analyses were performed to assess the effect of parameter uncertainty on model robustness.

**Results:**

When used in conjunction with SoC, finerenone extended time free of CV events and renal replacement therapy by respectively 0.30 and 0.31 life years compared to SoC alone, resulting in an extension of 0.20 quality-adjusted life years (QALYs). The reduction in renal and CV events led to a €6136 decrease in total lifetime costs per patient compared to SoC alone, establishing finerenone as a dominant treatment option. Finerenone in addition to SoC had a 83% probability of being dominant and a 93% probability of being cost-effective at a willingness-to-pay threshold of €20,000.

**Conclusion:**

By reducing the risk of CKD progression and CV events, finerenone saves costs to society while gaining QALYs in patients with T2D and advanced CKD in the Netherlands.

**Supplementary Information:**

The online version contains supplementary material available at 10.1186/s12933-023-02053-6.

## Background

Type 2 diabetes (T2D) affects over one million people in the Netherlands in total [1]. T2D can lead to vascular and neuropathic damage which increases the risk of chronic conditions, including chronic kidney disease (CKD) [2]. National data suggest that approximately 36% of Dutch patients with T2D run a mildly to strongly increased risk of CKD [1]. Moreover, at any stage, CKD is associated with a higher risk of progression to end-stage kidney disease (ESKD), renal death, and cardiovascular (CV) morbidity, including heart failure (HF), myocardial infarction (MI), stroke, and CV death [1].

High demand for first- and second-line care in CKD and T2D leads to significant healthcare expenses. In the Netherlands in 2019, the total expenditure associated with diabetes was €1.3 billion, or 1.2% of total healthcare costs [3]. A significant proportion of costs was related to general practitioner (GP) visits (i.e., approximately 31%) and hospital care (i.e., approximately 14%) [1, 4]. The costs for CKD in that same year were estimated at €805 million [3].

In general, the treatment algorithm recommended in the guidelines of the Dutch College of General Practitioners (*Nederlands Huisartsen Genootschap,* NHG) and Dutch Society of Internal Medicine (*Nederlandse Internisten Vereniging,* NIV) can be considered the standard of care (SoC) in the Netherlands [5–7]. According to those guidelines, patients with stages 3–4 CKD with an estimated glomerular filtration rate (eGFR) of ≥ 25– < 60 mL/min/1.73 m^2^ and moderately or severely increased albuminuria should be treated with angiotensin-converting enzyme (ACE) inhibitors or angiotensin receptor blockers (ARBs), if possible [5–7]. Statins and platelet aggregation inhibitors can be used to lower the risk of CV events. For patients with CKD associated with T2D, regulation of blood glucose is important. Blood glucose-lowering treatments include metformin, sulfonylurea, sodium-glucose cotransporter-2 (SGLT2) inhibitors, glucagon-like peptide 1 (GLP-1) receptor agonists, dipeptidyl peptidase-4 (DPP-4) inhibitors, and insulin therapy [6, 7]. Previously, SGLT2 inhibitors were solely administered as glucose-lowering treatments, but their treatment indication was extended in 2021 as they showed significant effects in patients with CKD and/or CV risk [8]. The recent NIV and NHG guidelines recommend SGLT2 inhibitors for the reduction of CKD progrend CV risk in patients with CKD associated with T2D with a very high risk for CV diseases [9].

Since June 2022, the selective nonsteroidal mineralocorticoid receptor (MR) antagonist finerenone has been reimbursed in the Netherlands for adult patients with CKD associated with T2D [10]. The FIDELIO-DKD trial assessed the effect of adding finerenone to SoC versus SoC on renal outcomes and time to CV events in more than 5,600 patients with T2D and predominantly stage 3 or 4 CKD with moderately or severely elevated albuminuria for an average follow-up duration of 2.6 years [13]. The SoC in the FIDELIO-DKD trial consisted mainly of ACE inhibitors or ARB treatment, and a relatively small proportion of patients (i.e., 6.2% in the intention to treat [ITT] population) was also treated with SGLT2 inhibitors. Compared to the placebo, the addition of finerenone to SoC significantly reduced the number of renal events and CV events in patients with CKD associated with T2D. [13, 14].

To ensure efficient resource allocation, given the wide range of treatments for patients with CKD associated with T2D and the relatively significant healthcare expenditure, it is important to assess the cost-effectiveness of new treatments. Recently, the FINE-CKD model has been validated for estimating the cost-effectiveness of finerenone in patients with CKD associated with T2D [15, 16]. This study uses the FINE-CKD model populated with data from the FIDELIO-DKD trial to investigate the cost-effectiveness of finerenone in addition to SoC, compared with current SoC in patients with T2D and advanced CKD from a Dutch societal perspective, according to the Consolidated Health Economic Evaluation Reporting Standard 2022 (Cheers 2022) reporting guidance [13, 17].

## Methods

The FINE-CKD model was used to calculate the cost-effectiveness ratio (ICER) of finerenone in combination with SoC compared to SoC in patient that represent the FIDELIO DKD trial. The FINE-CKD is a Markov model developed in Microsoft Excel 2016 (Redmond WA, USA) [15, 16].

### Patient population

The FIDELIO-DKD trial predominantly included patients with CKD stage 3 or 4 with moderately or severely elevated albuminuria associated with T2D (Table 1) [13]. Dutch patients with T2D are 68.5 years old on average when diagnosed with CKD, which is comparable with the average age in the FIDELIO trial [1, 13].

**Table 1:** Patient characteristics of the FIDELIO-DKD ITT population [13]

Parameter	Value at baseline (95% CI)
Age	65.6 (47.8–83.4)
Proportion male (%)	70.2% (69.0–71.4%)
Proportion with CKD 1/2 (%)	11.6%^a^
Proportion with CKD 3a/b (%)	76.2%^a^
Proportion with CKD 4 (%)	12.3%^adistri^
Proportion with CKD 5 (%)	0.0%
Proportion with ESKD and dialysis (%)	0.0%
Proportion with ESKD and transplantation (%)	0.0%

*CI* confidence interval, *CKD* chronic kidney disease, *ESKD* end-stage kidney disease

^a^Proportions of CKD stage are dependent on each other and were varied in sensitvity analyses by using a Dirichlet distribution

### Interventions

Finerenone was added to SoC and compared to SoC alone. The SoC was based on the weighted average of background treatment over the time horizon in the FIDELIO-DKD trial (Additional file 1). Patients used a mix of ACEIs, ARBs, Beta-blockers, diuretics, calcium antagonists, and glucose-lowering therapies. As seen in the FIDELIO-DKD trial, patients were anticipated to carry a discontinuation risk of 0.03 per cycle for finerenone treatment. In addition, after initiation of RRT, all patients were assumed to discontinue finerenone treatment, and 25% of patients to discontinue SoC treatment due to the increased risk for hyperkalemia.

### Model structure

**Fig. 1:**
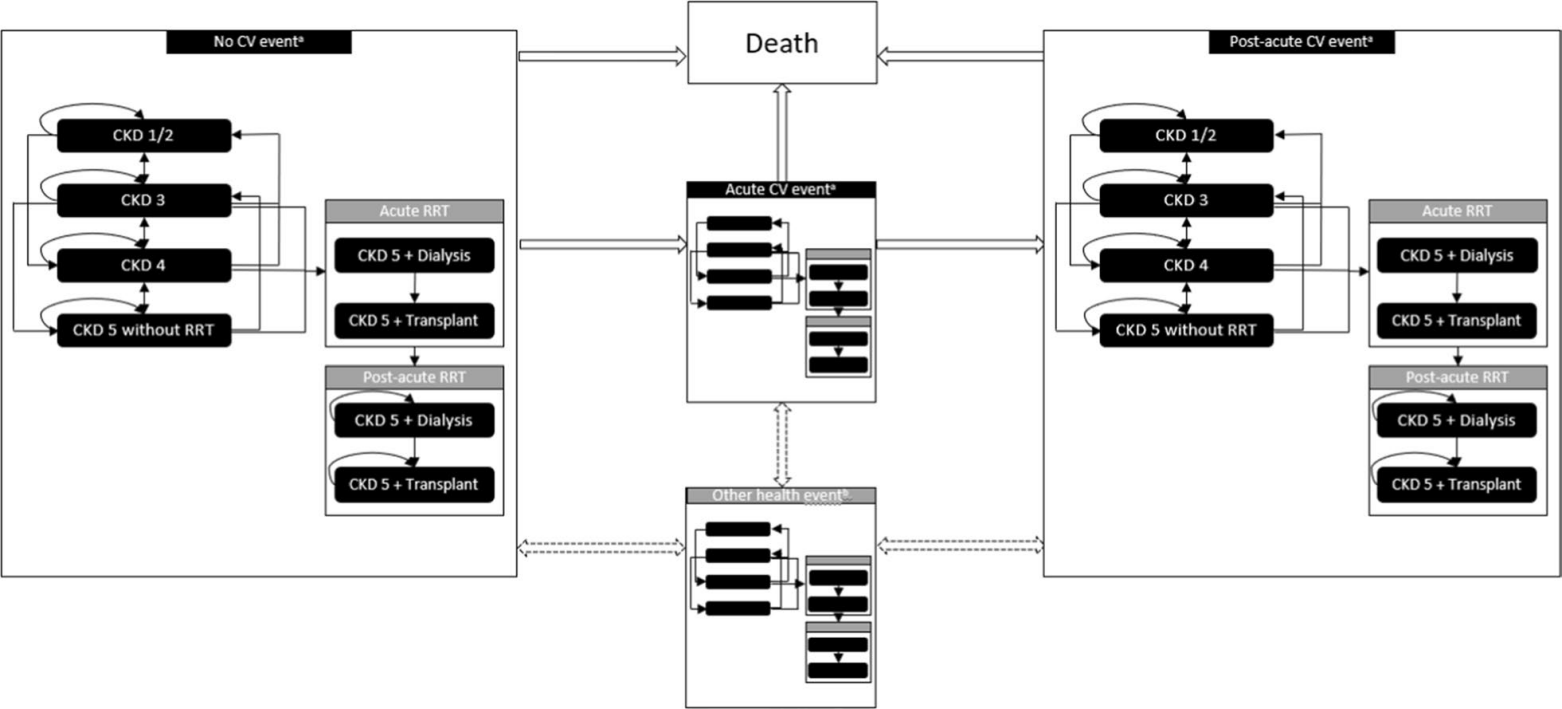
Model structure Key: This figure depicts the modelled CKD in detail. The left panel depicts the CKD health states in which patients start the model. Patients can experience CKD progression and/or experience a CV event. When patients experience a CV event, they move to the acute CV event panel for one cycle and to the post-acute CV panel for the rest of the model duration. OHEs can occur in any depicted health state. A patient can move to the death health state from every health state. ^a^CV events include a non-fatal stroke, non-fatal MI, and hospitalization for HF. ^b^OHEs include a subsequent CV event, hyperkalemia leading to hospitalization, hyperkalemia not leading to hospitalization, and a new onset of atrial fibrillation/atrial flutter. *CV* cardiovascular, *CKD* chronic kidney disease, *HF* heart failure, MI myocardial infarction, *OHE* other health event, *RRT* renal replacement therapy

The modelled discrete health states were defined in accordance with the CKD stage and history of CV events (Fig. 1). The model outcomes are validated to adequately reflect the clinical data and outcomes of other models [16]. Four stages of CKD health state progression were considered: CKD 1/2, CKD 3a/b, CKD 4, and CKD 5 without renal replacement therapy (RRT). Two health states for CKD5 or ESKD patients with RRT were considered: CKD 5 and dialysis, and CKD 5 and transplantation. In the absence of differentiated costs and outcomes, distinguishing between CKD 1 and CKD 2 or CKD 3a and 3b patients proved impossible. Patients resembled the trial population and entered the model in one of the CKD stages without CV events [13]. Patients remained in the same CKD stage for a cycle duration of four months or moved to either a more or a less advanced CKD stage while, at the same time, experiencing their first modelled CV event (non-fatal MI, non-fatal stroke, hospitalization for HF, or death). Transition probabilities were based on patient-level data of the FIDELIO-DKD trial; therefore, the model was not limited to CKD deterioration, and patients could move through multiple CKD stages per cycle. Once a CV event occurred, patients moved to the acute CV event health state for one cycle to account for the short-term impact of the CV event and then moved to the post-acute CV event health state for the rest of the model duration.

Apart from CKD progression and CV events, other health events (OHEs) were included in the model to account for additional relevant clinically meaningful outcomes seen in the FIDELIO-DKD trial (Additional file 2) [13]. OHEs included a subsequent CV event, hyperkalemia leading to hospitalization, hyperkalemia not leading to hospitalization, and a new onset of atrial fibrillation/atrial flutter. The risk of OHEs was dependent on the history of CV events; however, to reduce model complexity, OHEs were not modelled as discrete health states and did not affect downstream risks. Instead, their only impact was on costs and utility for one cycle length. Similar to acute CV events, OHEs were modelled for one cycle length.

In line with the observed mortality in the FIDELIO-DKD trial, three different reasons for death were implemented in the model (i.e., CV, renal, and mortality from other causes [not CV or renal related]) [13]. The 4 month cycle length was consistent with the primary endpoints in the FIDELIO-DKD trial and was supported by the fact that CKD associated with T2D is a chronic disease [13]. The Dutch guideline for economic evaluations in healthcare recommends a lifetime time horizon with a 100 year maximum age [18].

### Transition probabilities

The transition of patients between health states was dependent on the probability of CKD progression, CV events, death from renal and CV causes, and OHEs (Table 2) [13]. These probabilities were derived from patient-level data of the FIDELIO-DKD-ITT population [13]. While the FIDELIO-DKD trial was designed to assess composite renal and cardiovascular outcomes, demonstrating a significant positive effect of finerenone, different HRs were used to incorporate the influence of finerenone on the model treatment pathways to prevent double counting. The HRs for CV events, dialysis, CV, and renal death of the FIDELIO-DKD trial were used to adjust for the effect of finerenone on CKD progression and CV events (Table 3) [13]. Additionally, we used the HR for the sustained eGFR decrease < 15 mL/min/1.73 m^2^ to adjust for the effect of finerenone on progression to CKD 5. As the median follow-up duration in the FIDELIO-DKD trial was 2.6 years, HRs to account for the longer-term risk of a CV event (Additional file 3), increased risk of renal and CV mortality, and occurrence of the first modelled CV event (Additional file 3) were derived from literature [19–21]. Mortality from causes other than renal and CV events was retrieved from Dutch Central Bureau of Statistics data and adjusted for the proportion of deaths caused by CV events and CKD (Additional file 4) [22].

**Table 2:** Transition probabilities: CKD progression and first modelled CV event and OHE probabilities [13]

	CKD1/2	CKD3	CKD4	CKD5 w/o dialysis	Dialysis (acute)	Dialysis (post-acute)	Kidney Transplant (acute)	Kidney Transplant (post-acute)
Transition probabilities CKD progression for patients receiving SoC^a^ [13]								
CKD1/2	0.6696	0.3268	0.3600					
CKD3	0.0350	0.8705	0.0931	0.0011	0.0002			
CKD4	0.0012	0.1400	0.8043	0.0448	0.0096			
CKD 5 w/o dialysis		0.0135	0.0889	0.7143	0.1779		0.0054	
Dialysis (acute)						1.0000		
Dialysis (post-acute)						0.9921	0.0079	
Kidney transplant (acute)								1.0000
Kidney transplant (post-acute)								1.0000

	CKD1/2	CKD3	CKD4	CKD5 w/o dialysis	Dialysis (acute)	Dialysis (post-acute)	Kidney Transplant (acute)	Kidney Transplant (post-acute)
Transition probabilities CKD progression for patients receiving SoC and Finerenone^a^ [13]								
CKD1/2	0.6305	0.3665	0.0030					
CKD3	0.0269	0.8770	0.0949	0.0009	0.0002			
CKD4	0.0016	0.1548	0.7982	0.0371	0.0083			
CKD 5 w/o dialysis		0.0075	0.1267	0.7045	0.1559		0.0054	
Dialysis (acute)						1.0000		
Dialysis (post-acute)						0.9921	0.0079	
Kidney transplant (acute)								1.0000
Kidney transplant (post-acute)								1.0000

	CKD1/2	CKD3	CKD4	CKD5 w/o dialysis	Dialysis (acute)	Dialysis (post-acute)	Kidney Transplant (acute)	Kidney Transplant (post-acute)
Transition probabilities for CV events per CKD stage [13]								
Any CV event probability^b^	0.0119	0.0127	0.0157	0.0208	0.0208	0.0208	0.0157	0.0157
CV death	0.0062	0.0052	0.0081	0.0157	0.0191	0.0191	0.0081	0.0081
Renal death	0.000	0.000	0.000	0.0001	0.000	0.000	0.000	0.000
Probabilities of OHEs [13]	**No CV events**				**CV event**			
Subsequent CV event	–				7.32%			
Hyperkalaemia leading to hospitalization	0.07%				0.38%			
Hyperkalaemia not leading to hospitalization	1.63%				2.35%			
New onset of atrial fibrillation	0.35%				2.16%			

*CKD* chronic kidney disease, *CV* cardiovascular, *eGFR* estimated glomerular filtration rate, *HR* hazard ratio, *MI* Myocardial infarction, *OHE* Other health events, *SoC* standard of care, *w/o* without

^a^The transition probabilities of CKD progression were based on end points that were measured every four months in the FIDELIO-DKD trial with a median follow-up of 2.6 years. For patients that received finerenone, the probability of transitioning to CKD 5 was adjusted with the HR for the onset of eGFR decrease < 15 mL/min/1.73 m^2^ sustained over at least 4 weeks, and the probability of transition to dialysis was adjusted with the HR for progression to dialysis

^b^CV events included non-fatal MI, stroke, or hospitalization for heart failure

**Table 3:** HRs used to reflect the effectiveness of finerenone [13]

Description	HR Finerenone + SoC vs. SoC (95%CI)
The onset of eGFR decrease < 15 mL/min/1.73 m^2^ sustained over at least 4 weeks	0.82 (0.67–1.01)
Progression to dialysis	0.87 (0.67–1.12)
Progression to kidney transplant	1.00^a^ (1.00–1.00)
CV death	0.86 (0.68–1.08)
Renal death, CKD 5 w/o RRT	1.03 (0.15–7.31)
First modelled CV event^b^	0.87 (0.74–1.02)
Subsequent CV event^b^	0.95 (0.70–1.30)
Hyperkalemia leading to hospitalization	2.71 (1.60–4.60)
Hyperkalemia not leading to hospitalization	1.92 (1.67–2.21)
New onset of atrial fibrillation/atrial flutter	0.71 (0.53–0.94)

*CKD* chronic kidney disease, *CV* cardiovascular, *eGFR* estimated glomerular filtration rate, *HR* hazard ratio, *RRT* renal replacement treatment, *SoC* standard of care

^a^No difference between treatments was assumed; validated with clinical expert input

^b^CV events include non-fatal MI, stroke, or hospitalization for heart failure

### Utilities

Quality-adjusted life years (QALYs) were calculated to express the effect of finerenone on life years gained corrected for quality of life. Utility values measure the health-related quality based on preference values attached to the patient’s health status. Utility values were scaled between 0 (equal to death) and 1 (equal to perfect health). Utility values were derived from the FIDELIO-DKD trial for the health states and OHEs with the EuroQol five-dimension questionnaire (EQ-5D, specifically the five-level EQ-5D-5L) using a multilevel mixed repeated measurements model and the Dutch EQ-5D value set [13, 23]. As the number of patients who experienced RRT in the FIDELIO-DKD trial was low, a systematic literature review (SLR) was undertaken to estimate the disutility during dialysis and transplantation [24]. Utility values were adjusted for age, using the population norms for the Netherlands [25]. Additional file 5 presents the baseline utility and utility decrements for the different health states used in the model. All utilities were discounted at 1.5% per year, following Dutch guidelines for economic evaluations in healthcare [18].

### Costs

Costs within the healthcare system (i.e., medical costs) and costs for patients and caregivers (i.e., indirect non-medical costs) were included in the model in accordance with Dutch guidelines [18]. They were mostly based on Dutch literature and inflated to March 2023 prices [26], with an applied discount rate of 4% per year [18].

#### Costs within the healthcare system

Drug costs for finerenone and the current SoC were based on list prices per defined daily dose (Table 4, additional file 1) [13, 27]. We assumed that finerenone treatment discontinuation impacted both costs and effects by considering the same transition probabilities as patients treated with SoC. Treatment discontinuation of SoC only impacted costs. All treatment discontinuation assumptions were validated by clinical experts.

**Table 4:** Overview of model inputs for costs within the healthcare system

Parameter	Value	Reference
Medication costs finerenone (per day)	€2.00 (excl. VAT)	Medicijnkosten.nl and data on file [27, 33]
Average cost of SoC (per DDD)	€2.32	Farmaocotherapeutisch Kompas [34], GIP databank [32], Data on file [27], Additional file 1
CKD 1/2 (per cycle)^a^	€74	NHG guideline [5], Dutch costing manual [35] Schrauben et al. [36], NZA price index [37], Additional file 6
CKD 3 (per cycle)^a^	€140	NHG guideline [5], Dutch costing manual [35] Schrauben et al. [36]., NZA price index [37], Additional file 6
CKD4^a^	€301	NHG guideline [5], Dutch costing manual, Schrauben et al. [36]., NZA price index [37], Additional file 6
CKD 5 without RRT (per cycle)^a^	€467	NHG guideline [5], Dutch costing manual [35] Schrauben et al. [36]., NZA price index [37], Additional file 6
Haemodialysis and peritoneal dialysis (per cycle)	€39,179	Mohnen et al. [28]
Acute kidney transplant (one cycle)	€24,523	Mohnen et al. [28]
Post-acute kidney transplant (per cycle)	€6449	Mohnen et al. [28]
Acute MI (one cycle)	€4038	Van Schoonhoven et al. [29]
Post-acute MI (post-acute) (per cycle)	€748	Van Schoonhoven et al. [29]
Acute IS and ICH stroke (one cycle)	€11,169	Van Schoonhoven et al. [29]
Post-acute IS and ICH stroke (per cycle)	€3157	Van Schoonhoven et al. [29]
Acute hospitalisation for heart failure (one cycle)	€2578	Van Schoonhoven et al. [29]
Post-acute hospitalization for heart failure (per cycle)	€274	Van Schoonhoven et al. [29]
Subsequent CV event (per event)	€4852	Van Schoonhoven et al. [29]
Hyperkalemia (not leading to hospitalization) (one cycle)^b^	€306	GIP databank [32], assumption: weighted average of calcium polystyrene sulfonate, sodium polystyrene sulfonate, sodium zirconium cyclosilicate, or patiromer
Hyperkalemia (leading to hospitalization) (one cycle)^c^	€2873	Dutch costing manual [35], DBC for dialysis: 140301010 assumption
Atrial fibrillation/atrial flutter (one cycle)	€1160	Ringborg et al. [31]

*CBS* Central Agency for Statistics, *CKD* chronic kidney disease, *CV* cardiovascular, *DDD* daily defined dose, *eGFR* estimated glomerular filtration rate, *GP* general practitioner, *ICH* intracerebral haemorrhage, *IS* ischaemic stroke, *MI* myocardial infarction, *NHG* Dutch Society of Internal Medicine, *RRT* renal replacement therapy, *SoC* standard of care, *VAT* value added tax

^a^Resource used per CKD health state included visits to the GP, outpatient visits, eGFR and albuminuria assessments, treatment with an ACE inhibitor, an influenza vaccine, and risk for a hospital admission unrelated to CV outcomes

^b^Patients experiencing hyperkalaemia without hospitalization were assumed to use calcium polystyrene sulfonate, sodium polystyrene sulfonate, calcium polystyrene sulfonate, sodium zirconium cyclosilicate, patiromer

^c^Patients experiencing hyperkalaemia with hospitalization were in 80% of the cases assumed to have been admitted to hospital for 3 days. In 20% of cases the patients were admitted to an intensive care unit for one day and experienced regular hospital admission for 2 days. 10% of patients experienced acute dialysis

Health state costs associated with CKD 1/2, CKD 3a/b, CKD 4, and CKD 5 were calculated using a bottom-up approach validated by clinical experts. Resource use was based on CKD progression as well as the NHG and NIV guidelines, incorporating visits to the GP, outpatient visits, eGFR and albuminuria assessments, treatment with an ACE inhibitor, an influenza vaccine, and risk for a hospital admission unrelated to CV outcomes [5] (Additional file 6). Costs of ESKD with dialysis or transplantation, CV events, and OHEs were based on the literature and adjusted for the four-month cycle [28–31]. For dialysis and transplantation, both direct and indirect medical costs were considered based on Dutch health insurance claims [28]. Indirect costs included healthcare, medication, medical devices, and transportation (Additional file 7). It was assumed that patients with mild hyperkalemia (not leading to hospitalisation) were treated with either calcium polystyrene sulfonate for 40 days, sodium polystyrene sulfonate for 40 days, sodium zirconium cyclosilicate for 106 days, or patiromer for a full cycle, according to the Dutch Medicine and Resource Information Project (*Genees- en hulpmiddelen Informatie Project*, GIP) databank [32]. It was further assumed that patients with severe hyperkalaemia were admitted to an intensive care unit for one day and then transferred to a general ward for a two-day admission (i.e., 20% of all admitted cases). Additionally, 10% of all patients experienced acute dialysis. The other 80% who require hospitalization were admitted to a general ward for three days on average.

#### Costs for patients and caregivers

The base case analysis incorporated costs for informal care and productivity losses, both based on literature and expert opinion (Table 5). Due to the low estimated impact, travel costs were not factored in. Productivity losses and informal care were considered during CKD stages 3–5, acute and post-acute CV events, dialysis, and kidney transplants for patients below the Dutch retirement age (i.e., 67 years) [38–40]. To calculate productivity losses, the friction cost method was used, as outlined in the Dutch costing guideline [35]. All productivity losses were valued at the hourly rate and vacancy data stemming from 2022 [41]. After initiation of dialysis and transplantation, a certain percentage of patients was estimated to be on long-term sick leave [40]. Although the friction method indicated that a vacancy in 2023 should be filled in approximately 20 weeks, our model structure allowed us to incorporate a maximum duration of sick leave for a full cycle (i.e., 12 weeks) in acute dialysis and transplantation health states [35]. In addition to sick leave, short-term production losses were taken into account for each stage of CKD progression, dialysis and transplantation, CV events and OHEs [38, 40]. Short-term productivity losses were adjusted to the labour participation rate found in Dutch CKD patients [42]. The impact of informal care was estimated by valuing the hours of informal care a patient received per cycle at the home care replacement rate (i.e., hourly wage informal care) based on the Dutch costing manual [35]. In case of the absence of informal care data, we applied the same ratio for informal care during both the acute and post-acute states, as observed in productivity losses.

**Table 5:** Overview of the model inputs for costs incurred by patients and caregivers

Parameter	Value	References
Hourly wage patient	€35	CBS statline [41]
Opportunity costs informal care	€18	Dutch costing manual [35]
Average labour participation wage (CKD 1/2)	69%	CBS [42]
Average labour participation wage (CKD 3 to5)	68%	Alma et. al[40]
Average labour participation wage (Dialysis)	52%	Alma et. al. [40]
Average labour participation wage (Transplantation)	64%	Alma et. al. [40]
Average working hours per week (Dialysis)	24 h per week	Alma et. al. [40]
Average working hours per week (Transplantation)	28 h per week	Alma et. al. [40]
Age at retirement	67 years	CBS [42]
Friction days	85 days	Dutch costing manual [35]
Costs for patients and caregivers—resource use		
CKD 3a/b	Short term productivity loss: 1.8 h per day	Alma et. al. [40] Expert opinion
	Informal care: 0 days	
CKD 4	Short term productivity loss: 1.8 h per day	Alma et. al. [40] Expert opinion
	Informal care: 4 days	
CKD 5 (without RRT)	Short term productivity loss: 1.8 h per day	Alma et. al. [40] Expert opinion
	Informal care: 5 days	
Acute dialysis	Percentage of patients on sick leave: 23% Short term productivity loss: 3.2 h per day	Alma et. al. [40] De Vries et. al. [39] and confirmed by expert opinion
	Informal care: 15 days	
Post-acute dialysis	Short term productivity loss: 3.2 h per day	Alma et. al. [40] De Vries et al. [39] and confirmed by expert opinion
	Informal care: 15 days	
Acute kidney transplantation	Percentage of patients on sick leave: 58% Short term productivity loss: 2 h per day	Alma et. al. [40] De Vries et. al. [39] and confirmed by expert opinion
	Informal care: 15 days	
Post-acute kidney transplantation	Short term productivity loss: 2 h per day	Alma et. al. [40] De Vries et. al. [39]
	Informal care: 0 days	
Acute MI	Productivity losses: 20 days per cycle	Kotseva et. al. [38] Assumption validated with expert
	Informal care: 4 days	
Acute stroke	Productivity losses: 19 days per cycle	Kotseva et. al. [38] Assumption validated with expert
	Informal care: 4 days	
Acute hospitalization for heart failure	Productivity losses: 20 days per cycle	Kotseva et. al. [38] Assumption validated with expert
	Informal care: 4 days	
Post-acute MI	Productivity losses: 0.7 days per cycle	Kotseva et. al. [38] Assumption validated with expert
	Informal care: 0.02 days per cycle	
Post-acute stroke	Productivity losses: 3 days per cycle	Kotseva et. al. [38] Assumption validated with expert
	Informal care: 0.7 days	
Post-acute hospitalization for heart failure	Productivity losses: 3 days per cycle	Kotseva et. al. [38] Assumption validated with expert
	Informal care: 0.6 days	

*CBS* Central Agency for Statistics, *CKD* chronic kidney disease, *CV* cardiovascular, *DDD* daily defined dose, *Egfr* estimated glomerular filtration rate, *IS* ischaemic stroke, *MI* myocardial infarction;

### Analyses

#### Base case analysis

In the base case, the model calculates the ICER for a willingness-to-pay (WTP) threshold of €20,000 (Table 6). The Dutch guidelines for economic evaluations in healthcare recommend a WTP threshold of €20,000 for a proportional disease shortfall of 0.10–0.40 QALY and a WTP threshold of €50,000 for a proportional disease shortfall of 0.40–0.60 QALY [43]. An estimated disease shortfall of 0.47 QALY was determined and since this value is considered low within the €50,000 WTP threshold, a conservative approach was taken, and the €20,000 WTP threshold was applied [43]. In addition to the life time horizon, the incremental costs were calculated for a time horizon of 1 to 34 years. Our analysis was described using the CHEERS reporting guidance for health economic evaluations (Additional file 8) [17].

**Table 6:** Overview of base case model characteristics

Parameter	Input
Patient population	Based on FIDELIO-DKD (T2D and CKD 2–4 with moderately and severely elevated albuminuria) [13, 35]
Interventions	Finerenone on top of standard of care vs. standard of care
Time horizon	Lifetime
Cycle length	4 months
Discounting	Costs: 4.0%, QALYs: 1.5% [35]
Perspective	Societal (including costs within the healthcare system and indirect costs for patients and caregivers) [35] Healthcare
Outcomes	ICER
WTP-threshold	€20,000

*CKD* chronic kidney disease, *ICER* incremental cost-effectiveness ratio, *QALY* quality-adjusted life-year, WTP willingness-to-pay threshold

#### Sensitivity analysis

The deterministic sensitivity analysis (DSA) was performed to assess the impact of the individual input parameters on the ICER by varying them between the lower and upper bounds of their confidence intervals (CIs), which were set at 2.5% and 97.5%, respectively. In addition, a probabilistic sensitivity analysis (PSA) was conducted as extension on the base case analysis to assess the model’s robustness, given the uncertainty around input parameters. That is, the PSA provides a range of results reflecting model uncertainty, whereas the base case assumes certainty around all selected parameters. Input parameters were simultaneously varied across 1000 simulations within their respective 95% CIs. The parameters in the analysis were varied with their respective distributions (normal, beta, gamma, Dirichlet). A standard error of 25% from the deterministic value was applied when the standard error or 95% CI were not available. An overview of all the included parameters, along with their respective CIs and distributions, is presented in Additional file 9.

#### Scenario analyses

Scenario analyses were performed to establish the impact of several input and model assumptions (Additional file 10). The time horizon was set to ten years and discount rates were varied in line with the Dutch guidelines of economic evaluations. In addition, to assess the impact of patient’s age at baseline, scenarios were performed in which patients were respectively 45, 55, and 68.5 years (i.e., the latter the average age of patients with T2D at diagnosis for CKD) at model initiation. Moreover, the impact of different sources of utility data were separately analysed. The number of patients with more advanced CKD and RRT in the FIDELIO-DKD trial was low [13]; therefore, in the base case analysis, a combination of utility data derived from the FIDELIO-DKD trial data and literature was used to estimate the utility values of patients who experienced dialysis or transplantation. To account for the uncertainty in the utility data, three additional scenario analyses were performed. In the first scenario, solely data from the FIDELIO-DKD trial was considered [13]. Subsequently, a scenario with solely utility data retrieved from the systematic literature review was performed [24, 44, 45]. An additional scenario analysis was performed using utility data previously validated by the Dutch National Healthcare Institute [46]. Additional file 5 presents the utility data incorporated in each scenario.

Our model considered all (in) direct costs related to dialysis and transplantation (e.g., healthcare, transportation, and medication). As indirect costs are a major part of the total cost related to dialysis and transplantation (18–19%), a scenario was run where only direct dialysis and transplantation costs were considered to assess the impact of indirect costs. In the base case, productivity losses were estimated with by the use of various literature sources. To address potential uncertainties in the methodology, a scenario analysis was conducted using alternative literature sources [38, 39, 47].

## Results

### Base case analysis

The base case results show that treatment with finerenone and SoC incurred 9.40 life years and 7.05 QALYs per patient over a lifetime horizon, compared to 9.18 life years and 6.85 QALYs for patients treated with SoC over a lifetime (Table 7). This difference of 0.22 life years and 0.20 QALYs can be attributed to an increase of 0.30–0.31 life years without RRT or a CV event (Table 7).

**Table 7:** Costs, QALYs, and ICER per patient over a lifetime for finerenone + SoC and SoC

	Finerenone + SoC	SoC	Difference
Costs within the healthcare system			
Medication costs	€10,098	€6554	€3545
CKD treatment	€4272	€4125	€148
Dialysis	€57,650	€65,584	− €7935
Transplant	€2262	€2125	− €275
First CV event costs	€5843	€6134	− €291
Subsequent CV event	€1528	€1633	− €105
Hyperkalaemia leading to hospitalization	€159	€88	€70
Hyperkalaemia not leading to hospitalization	€192	€126	€66
New onset of atrial fibrillation	€168	€198	− €30
Indirect costs for patients and caregivers	€111,076	€12,4069	− €1329
Total costs (healthcare perspective)	€82,172	€86,978	− €4708
Total costs (societal perspective)	€93,248	€99,384	− €6136
Total effects (life-years without CV)	7.43	7.13	0.30
Total effects (life-years without RRT)	8.65	8.34	0.31
Total effects (life-years)	9.40	9.18	0.22
Total effects (QALY)	7.05	6.85	0.20
ICER (costs/QALY)	**Finerenone + SoC is a dominant treatment option**		

CKD chronic kidney disease, *CV* cardiovascular, *eGFR* estimated glomerular filtration rate, *ICER* incremental cost-effectiveness ratio, *MI* myocardial infarction, *QALY* quality-adjusted life year, *RRT* renal replacement therapy, *SoC* standard of care

From a societal perspective, treatment with finerenone and SoC is associated with total costs of €93,248 per patient over a lifetime, while treatment with solely SoC incurs total costs of €99,384. This implies a cost-reduction of €6136 per patient associated with the addition of finerenone to SoC. The largest cost reductions with finerenone occur in dialysis costs (€7935), costs for patient and caregiver (€1329), and first CV event costs (€291). Adding finerenone to SoC increases medication costs (€3545), CKD treatment costs (€148), costs related to hyperkalemia not leading to hospitalisation (€66), and costs related to hyperkalemia leading to hospitalisation (€70). A healthcare perspective (i.e., excluding the indirect non-medical cost) results in incremental costs of − €4708. The combination of increased QALYs and reduced costs associated with finerenone and SoC, compared to SoC alone, establishes finerenone as the dominant treatment option over SoC from a societal and healthcare perspective.

### Cost-savings over time

**Fig. 2:**
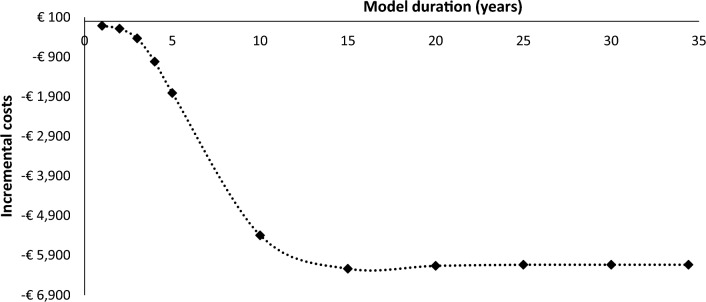
Incremental cost-savings over model duration of 1 to 34.4 (i.e., life-time) years

Figure 2 estimates the incremental costs over time. The cost-savings caused by finerenone treatment increase over time. However, even after 1 and 5 years of treatment, finerenone in combination with SoC saves respectively €115 and €1809, compared to SoC alone. The incremental costs decrease the most in the first 10 years, which indicates that finerenone prevents the most CV and renal events in this period. The incremental costs remain constant after 15 years until the life-time horizon (i.e., 34.4 years).

### Deterministic sensitivity analysis

**Fig. 3:**
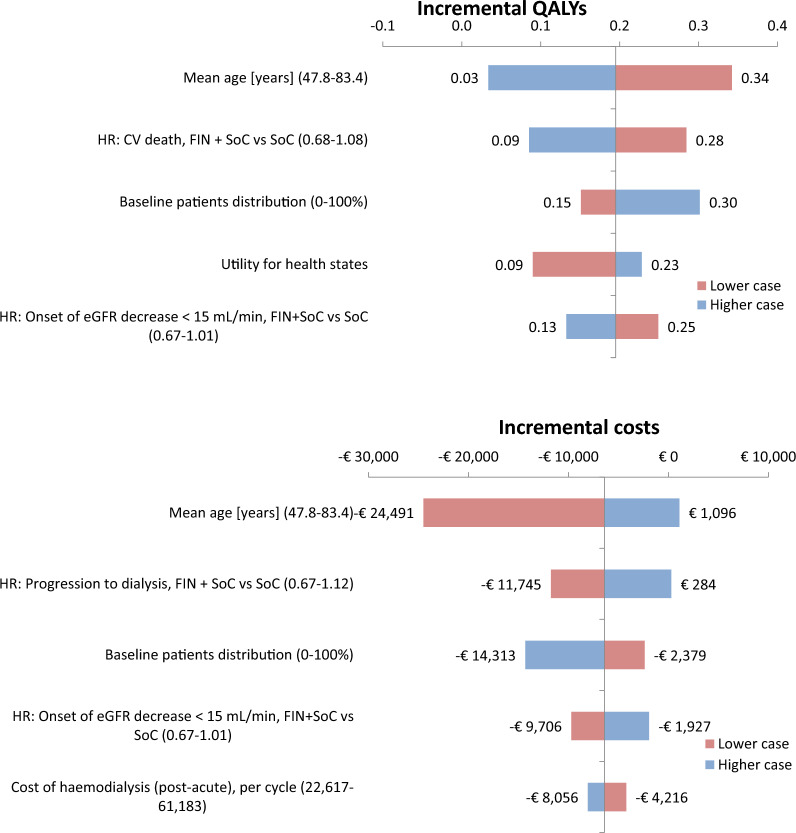
Tornado diagram presenting parameters with most influence on incremental QALYs an costs Key: The lower case presents the outcome for the 2.5% CI of the distribution. The higher case presents the outcome of the 97.5% CI of the distribution. *CKD* chronic kidney diseas, *CV* cardiovascular event; *FIN* finerenone; *HR* hazard ratio, SoC standard of care

The DSA shows that the analysis is most sensitive to the average age at baseline. Specifically, a reduction of the average age to the 2.5% CI (i.e., 47.8 years) leads to a 0.14 increase in QALYs and an €18,355 decrease in costs (Fig. 3). Other parameters that the incremental QALYs are sensitive to include HR for CV death (0.09**–**0.28), baseline patient distribution (0.15**–**0.30), utility for health states (0.09–0.23), and the HR for the onset of eGFR decreased < 15 mL/min (0.13**–**0.25). A lower HR for CV death, higher numbers of patients with more advanced CKD at baseline, and a lower HR for the onset of eGFR decrease < 15 mL/min contribute to higher incremental QALYs. However, the DSA results indicate that finerenone leads to an increase in incremental QALYs across all included CIs.

HRs that influence incremental costs include the HR for progression to dialysis (ranging from -€11,745 to €284) and the HR for the onset of eGFR decrease < 15 mL/min (ranging from − €9706 to -€1927) (Fig. 3). A lower HR for progression to dialysis and a lower HR for the onset of eGFR decrease < 15 mL/min lead to increased prevention of renal and CV events and thus, increase in cost savings for finerenone. However, finerenone remained cost-saving over the entire distribution for the onset of eGFR decrease < 15 mL/min. The incremental costs increased to €284 for the upper bound of the HR for progression to dialysis. Additionally, the incremental costs were sensitive to the baseline distribution of patients (ranging from − €14,313 to − €2379) and the costs of haemodialysis (ranging from − €8056 to − €4216). Considering that the incidence of renal and CV events is higher in patients with more severe CKD progression, the impact of finerenone becomes more pronounced, leading to increased cost savings.

### Probabilistic sensitivity analysis

**Fig. 4:**
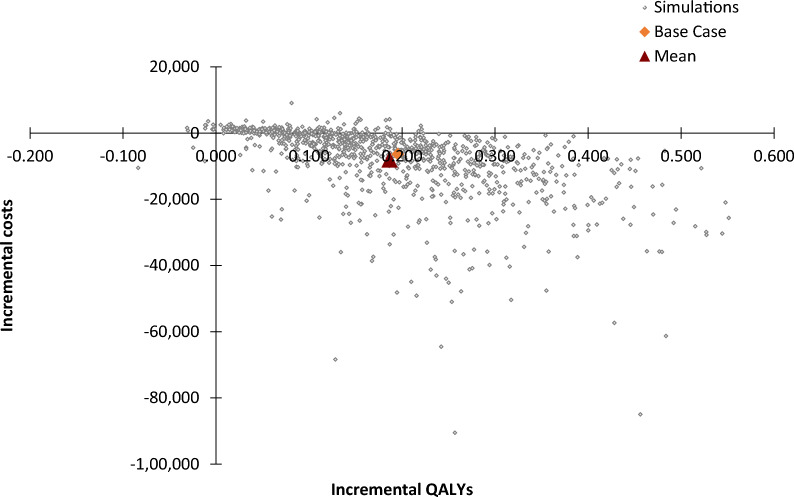
Cost-effectiveness plane Key: The orange mark represents the base case ICER. The red mark represents the average ICER that was found in the PSA. *ICER* incremental cost-effectiveness ratio, *PSA* probabilistic sensitivity analysis, *QALYs* quality-adjusted life years

**Fig. 5:**
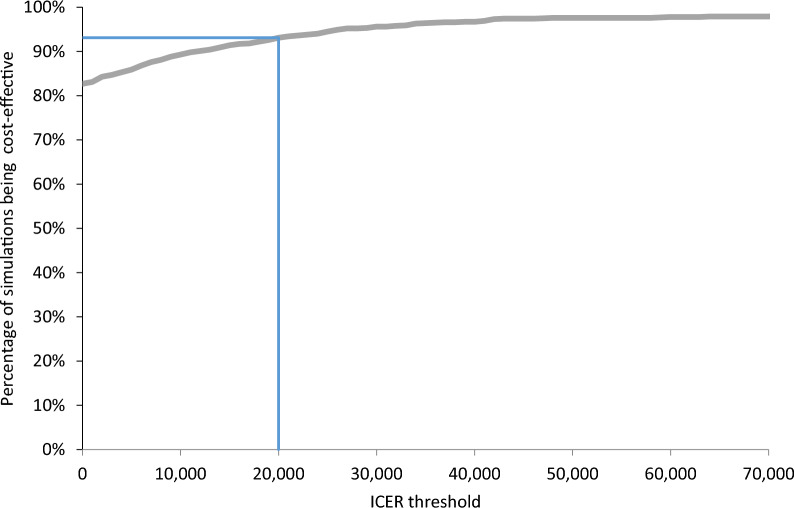
Cost-effectiveness acceptability curve. The blue line represents the WTP threshold of €20,000. *ICER* incremental cost-effectiveness ratio

The cost-effectiveness plane (Fig. 4) and cost-effectiveness acceptability curve (Fig. 5) present the outcomes of the PSA. The PSA shows that after considering the uncertainty around the input parameters, the outcome is consistent with the base case analysis (i.e., treatment with finerenone and SoC dominates treatment with SoC). The average incremental QALYs value is 0.19, and the average incremental costs value is − €7994. The lower and upper bounds of the incremental QALYs (2.5–97.5% CI) are 0.02 and 0.44, respectively. The lower and upper bounds of the incremental costs are − € 37,448 and €1989, respectively. The PSA outcomes present a cost-effectiveness plane covering all four quadrants, but adding finerenone to SoC has a probability of 82.5% being dominant. In addition, the cost-effectiveness acceptability curve shows that adding finerenone to SoC has a 93.1% probability of being cost-effective at a WTP threshold of €20,000.

### Scenario analysis

Finerenone gains QALYs and saves costs in all scenarios, and thus remains dominant in all scenarios (Table 8). A discount rate of 0% leads to a larger gain in QALYs (0.23) and more cost-saving (€8,133). In addition, a lower age at baseline (i.e., 45 or 55 years) increases incremental QALYs and incremental cost-savings. On the other hand, a higher age at baseline (i.e., 65.8 years), 10 year time horizon, and the inclusion of solely direct dialysis or different sources for productivity losses reduce the cost savings. Different sources for utility data impact the incremental QALYs by 0.1–0.3.

**Table 8:** Outcomes of the scenario analyses

Description	Incremental costs (discounted)	Incremental QALYs (discounted)	ICER (discounted)
Base case	− €6136	0.20	Dominant
The discount rate is 0% for both costs and effects	− €7957	0.23	Dominant
Utility data is based on literature retrieved in a systematic literature review	− €6136	0.19	Dominant
Utility data is based solely on trial data	− €6136	0.17	Dominant
Utility data is based on retrieved from the literature that was validated by the Dutch National Healthcare Institute	− €6136	0.19	Dominant
Patients are 45 years old at baseline	− €26,622	0.36	Dominant
Patients are 55 years old at baseline	− €17,733	0.29	Dominant
Patients are 65.8 years old at baseline (i.e., the average age of Dutch patients with T2D at CKD diagnosis)	− €5708	0.19	Dominant
The discount rate is 5% for both costs and effects	− €5766	0.14	Dominant
Time horizon 10 years	− €5396	0.11	Dominant
Solely direct dialysis costs are incorporated	− €4414	0.20	Dominant
Productivity losses are based on different sources	− €5545	0.20	Dominant

*CKD* chronic kidney disease, *ICER* incremental cost-effectiveness ratio, *QALYs* quality-adjusted life year, *T2D* Type 2 Diabetes

## Discussion

This study estimated the cost-effectiveness of adding finerenone to SoC in patients with advanced CKD associated with T2D. Adding finerenone to the current SoC increases incremental QALY for the patients and saves costs from both a societal and healthcare perspective. This is mainly attributable to the reduction in the number of dialyses, the number of CV events as well as the associated (in) direct medical costs and costs for patient and caregiver.

In the Netherlands, the annual direct and indirect medical costs of dialysis are considerable and range between €77,566 and €105,833 per patient [28]. A significant part of these costs can be attributed to indirect costs; the average annual indirect costs vary between €14,000–20,800 per patient due to transportation to dialysis centres, hospital care, mental health care, and additional medication [28]. Finerenone reduces the risk for progression to dialysis by 18% and subsequently, the reduction in dialysis costs is a significant contributor to the cost-saving effects of finerenone. This is also illustrated by the deterministic sensitivity analysis, in which the incremental costs ranged from -€11,475 to €284 across the CI of the HR for progression of dialysis. To account for the impact of indirect dialysis costs, an additional scenario analysis was performed, which showed that even when indirect dialysis costs were excluded, finerenone remained cost-saving (i.e., -€4,960 per patient) and dominant.

Several assumptions were made to optimally reflect clinical practice in our model. Although all assumptions were validated by clinical experts, they unavoidably brought uncertainty to the outcomes of this study. First, to reduce model complexity, it was assumed that patients were at risk of experiencing a maximum of one CV event per four-month cycle length. Moreover, OHEs did not affect the subsequent risk of CV events, CKD progression, or survival in the model. Both are conservative assumptions, and they may have led to an underestimation of the number of (prevented) CV events and therefore, an underestimation of the true effect of finerenone.

Ideally, all inputs for the transition probabilities would have been sourced from the FIDELIO-DKD trial [13]. However, considering that the model’s time horizon extends across a lifetime, and the median follow-up duration of the trial was 2.6 years, additional inputs were required. They were necessary to account for factors such as an increased risk of CV events after a certain age or increased mortality from other causes due to CKD progression. The DSA showed that variations in the HR value for increased risk of a CV event had minimal effect on the outcomes, indicating that the model’s results remained stable. While the model was more sensitive to the HR used to account for the increase in mortality from other causes due to more severe CKD, it is noteworthy that finerenone remained dominant over SoC across the entire range, from lower to upper CI bound.

The primary renal and secondary CV composite outcome in the FIDELIO-DKD trial found a statistically significant effect of finerenone [13]. To properly account for the effect of finerenone on the treatment patterns in the model, more specific HRs were used, of which some of the CIs included [13]. While it is generally preferable to focus solely on significant endpoints in the analysis, the inclusion of these HRs was essential to accurately represent the disease pathway of CKD and T2D. This approach aligns with the recommendation from the International Professional Society for Health Economics and Outcomes Research (ISPOR), which emphasizes the incorporation of all available data for key parameters, even when they do not meet conventional thresholds of statistical significance [48].The DSA demonstrated that finerenone continued to yield increased incremental QALYs and cost savings across nearly all CI ranges of the HRs. Additionally, the PSA demonstrated the robustness of the model. The FINE-CKD model was developed to follow the patient pathway of patients with CKD associated with T2D in the FIDELIO-DKD trial [15, 16]. Consequently, external validation was performed to predict the alignment between the model outcomes and the clinical data, and it was indicated that the FINE-CKD model reflected the event rates of the FIDELITY-ITT pooled analysis accordingly [15]. However, to understand if the model outcomes reflected real life, we also considered the real-world data of patients with CKD associated with T2D on SoC in the Netherlands. It shows that our model might have underestimated the number of CV events in comparison to real-world data and thus, the true effect of finerenone: our model found 1.03 CV events and/or deaths per patient for the model duration based on the FIDELI-DKD data while a Dutch real-world study found approximately 1.6 CV events and/or deaths per patient for the duration of the model [49]. Nevertheless, it is important to note that although the average age of these patients was similar, the CKD progression of the patients in this study differed from that of the FIDELIO-DKD population. Therefore, to further validate our findings, future studies should be used. The FINE-REAL study is an ongoing prospective observational study that investigates treatment patterns and safety in patients with CKD associated with T2D in the finerenone treatment. This study will also provide insight into how finerenone is being used in Dutch real-life practice [50]. Recent trials have shown that the SGLT2 inhibitors⁠—canagliflozin, dapagliflozin, and empagliflozin⁠—were also effective in reducing CKD progression, and CV events in patients with CKD [51–54]. A post-hoc analysis of the DAPA-CKD trial investigated the influence of baseline MR antagonists on the primary outcomes of dapagliflozin administration in patients with CKD, revealing that the efficacy of SGLT2 inhibitors remained unaffected by the presence of MR antagonists [55]. While the DAPA-CKD trial did not include any patients receiving the nonsteroidal MR antagonist finerenone, a post-hoc analysis of the FIDELIO-DKD trial further demonstrated that the impact of finerenone on CV and renal outcomes was independent of SGLT2 inhibitor use [56]. Given the increase in SGLT2 inhibitor use after recent trials and updated guidelines [9], additionally, the recently started CONFIDENCE study is generating more robust data by investigating the effectiveness and safety of finerenone in combination with empagliflozin [57]. Therefore, the CONFIDENCE study can contribute to insights into the cost-effectiveness of finerenone and SGLT2 inhibitor combination therapy in future studies.

In addition, our study analysed the cost-effectiveness of finerenone in patients who were represented in the FIDELIO-DKD trial (i.e., predominantly stage 3 or 4 CKD with moderately or severely elevated albuminuria associated with T2D) [13]. At the time of analysis, this was the only patient population for which finerenone had received European Medicine Agency (EMA) approval [58]. However, the indication of finerenone was recently extended to patients with less severe CKD associated with T2D [14]. In a future study, it will be valuable to estimate the cost-effectiveness of finerenone the entire indication population with T2D with the data of the FIDELITY-ITT pooled analysis [14, 59].

The main strength of this study is that the model structure was validated and represented the disease pathway of patients with CKD associated with T2D. The model is mostly based on clinical data, and all assumptions have been verified by clinical experts. Additionally, by including societal costs, the model captures the impact of chronic disease from the perspective of both healthcare and society.

## Conclusions

The deterministic and probabilistic analysis showed that treatment with finerenone reduces renal and CV events in patients with CKD associated with T2D in the Netherlands, resulting in an increase in QALYs and a reduction of healthcare and societal costs. The probabilistic analysis indicated a high probability of treatment with finerenone being a cost-saving, and at least a cost-effective, addition to SoC in this patient group.

## Supplementary Information

**Additional file 1:** Incorporated standard of care.

**Additional file 2:** Other health events rationale.

**Additional file 3:** Description of the calculation of transition probabilities for CKD progression and CV events, and corresponding input data.

**Additional file 4:** Description of the calculation of mortality and corresponding input data.

**Additional file 5:** Utility values incorporated in the base case and scenario analyses.

**Additional file 6:** Costs and resource allocation used to calculate the costs per CKD health state.

**Additional file 7:** Healthcare claims used to calculate the costs related to dialysis and transplantation.

**Additional file 8:** Consolidated Health Economic Evaluation Reporting Standards 2022 (CHEERS 2022) statement: reporting guidance for health economic evaluations.

**Additional file 9:** Overview of included parameters and their distributions that was used in the probabilistic sensitivity analysis.

**Additional file 10:** Overview of assumptions that were used in the model.

## Data Availability

All data analysed during this study are included in this published article and its supplementary information files. Some of the patient-level data analysed during current study are not publicly available due to privacy restrictions but are available from the corresponding author on reasonable request.

## Abbreviations

ACE: Angiotensin-converting enzyme
ARB: Angiotensin receptor blockers
CHEERS: Consolidated Health Economic Evaluation Reporting Standards
CKD: Chronic kidney disease
CV: Cardiovascular
CI: Confidence interval
DPP-4: Dipeptidyl peptidase-4
DSA: Deterministic sensitivity analysis
ESKD: End-stage kidney disease
EQ-5D: EuroQol five-dimension questionnaire
eGFR: Estimated glomerular filtration rate
GP: General practitioner
GLP-1: Glucagon-like peptide 1
HF: Heart failure
HR: Hazard ratio
ICER: Incremental cost-effectiveness ratio
ISPOR: International Professional Society for Health Economics and Outcomes Research
MI: Myocardial infarction
MR: Mineralocorticoid receptor
NHG: Dutch College of General Practitioners
NIV: Dutch Society of Internal Medicine
OHE: Other health event
PSA: Probabilistic sensitivity analysis
QALY: Quality-adjusted life-year
SGLT2: Sodium-glucose cotransporter-2
SoC: Standard-of-care
SLR: Systematic literature review
T2D: Type 2 diabetes
WTP: Willingness to pay
