# Correction: Cost‑effectiveness of finerenone in chronic kidney disease associated with type 2 diabetes in The Netherlands

**DOI:** 10.1186/s12933-025-02752-2

**Published:** 2025-05-15

**Authors:** Sara W. Quist, Alexander V. van Schoonhoven, Stephan J. L. Bakker, Michał Pochopień, Maarten J. Postma, Jeanni M. T. van Loon, Jeroen H. J. Paulissen

**Affiliations:** 1 Department of Health Sciences, University of Groningen, Groningen, The Netherlands; 2 Asc Academics, Groningen, The Netherlands; 3 Division of Nephrology, Department of Internal Medicine, University Hospital Groningen, Groningen, The Netherlands; 4 Assignity, Kraków, Poland; 5 Department of Economics, Econometrics and Finance, University of Groningen, Groningen, The Netherlands; 6 Value-XS, Houten, The Netherlands

**Correction to: Cardiovascular Diabetology (2023) 22:328** 10.1186/s12933-023-02053-6

In the original publication of this article [1], the authors identified typographical errors in Tables 2 and 7. In Table 2, the transition probability from CKD1/2 to CKD4 should be “0.0036” instead of “0.36”. In Table 7, the total indirect costs “€111,706 and €12,4069” was incorrect and should have been “€11,706 and €12,406”, though the difference between the two values remains correct. These corrections do not affect the methodology, results, or conclusion section.

**Table 2:** Transition probabilities: CKD progression and first modelled CV event and OHE probabilities [13]

	CKD1/2	CKD3	CKD4	CKD5 w/o dialysis	Dialysis (acute)	Dialysis (post-acute)	Kidney Transplant (acute)	Kidney Transplant (post-acute)
Transition probabilities CKD progression for patients receiving SoC^a^ [13]								
CKD1/2	0.6696	0.3268	0.0036					
CKD3	0.0350	0.8705	0.0931	0.0011	0.0002			
CKD4	0.0012	0.1400	0.8043	0.0448	0.0096			
CKD 5 w/o dialysis		0.0135	0.0889	0.7143	0.1779		0.0054	
Dialysis (acute)						1.0000		
Dialysis (post-acute)						0.9921	0.0079	
Kidney transplant (acute)								1.0000
Kidney transplant (post-acute)								1.0000

	CKD1/2	CKD3	CKD4	CKD5 w/o dialysis	Dialysis (acute)	Dialysis (post-acute)	Kidney Transplant (acute)	Kidney Transplant (post-acute)
Transition probabilities CKD progression for patients receiving SoC and Finerenone^a^ [13]								
CKD1/2	0.6305	0.3665	0.0030					
CKD3	0.0269	0.8770	0.0949	0.0009	0.0002			
CKD4	0.0016	0.1548	0.7982	0.0371	0.0083			
CKD 5 w/o dialysis		0.0075	0.1267	0.7045	0.1559		0.0054	
Dialysis (acute)						1.0000		
Dialysis (post-acute)						0.9921	0.0079	
Kidney transplant (acute)								1.0000
Kidney transplant (post-acute)								1.0000

	CKD1/2	CKD3	CKD4	CKD5 w/o dialysis	Dialysis (acute)	Dialysis (post-acute)	Kidney Transplant (acute)	Kidney Transplant (post-acute)
Transition probabilities for CV events per CKD stage [13]								
Any CV event probability^b^	0.0119	0.0127	0.0157	0.0208	0.0208	0.0208	0.0157	0.0157
CV death	0.0062	0.0052	0.0081	0.0157	0.0191	0.0191	0.0081	0.0081
Renal death	0.000	0.000	0.000	0.0001	0.000	0.000	0.000	0.000
Probabilities of OHEs [13]	**No CV events**				**CV event**			
Subsequent CV event	–				7.32%			
Hyperkalaemia leading to hospitalization	0.07%				0.38%			
Hyperkalaemia not leading to hospitalization	1.63%				2.35%			
New onset of atrial fibrillation	0.35%				2.16%			

*CKD* chronic kidney disease, *CV* cardiovascular, *eGFR* estimated glomerular filtration rate, *HR* hazard ratio, *MI* Myocardial infarction, *OHE* Other health events, *SoC* standard of care, *w/o* without

^a^ The transition probabilities of CKD progression were based on end points that were measured every four months in the FIDELIO-DKD trial with a median follow-up of 2.6 years. For patients that received finerenone, the probability of transitioning to CKD 5 was adjusted with the HR for the onset of eGFR decrease < 15 mL/min/1.73 m^2^ sustained over at least four weeks, and the probability of transition to dialysis was adjusted with the HR for progression to dialysis.

^b^ CV events included non-fatal MI, stroke, or hospitalization for heart failure

**Table 7:** Costs, QALYs, and ICER per patient over a lifetime for finerenone + SoC and SoC

	Finerenone + SoC	SoC	Difference
Costs within the healthcare system			
Medication costs	€10,098	€6,554	€3,545
CKD treatment	€4272	€4,125	€148
Dialysis	€57,650	€65,584	− €7,935
Transplant	€2262	€2,125	− €275
First CV event costs	€5843	€6,134	− €291
Subsequent CV event	€1528	€1,633	− €105
Hyperkalaemia leading to hospitalization	€159	€88	€70
Hyperkalaemia not leading to hospitalization	€192	€126	€66
New onset of atrial fibrillation	€168	€198	− €30
Indirect costs for patients and caregivers	€11,076	€12,406	− €1,329
Total costs (healthcare perspective)	€82,172	€86,978	− €4,708
Total costs (societal perspective)	€93,248	€99,384	− €6,136
Total effects (life-years without CV)	7.43	7.13	0.30
Total effects (life-years without RRT)	8.65	8.34	0.31
Total effects (life-years)	9.40	9.18	0.22
Total effects (QALY)	7.05	6.85	0.20
ICER (costs/QALY)	Finerenone + SoC is a dominant treatment option		

*CKD* chronic kidney disease,* CV* cardiovascular,* eGFR* estimated glomerular filtration rate,* ICER* incremental cost-effectiveness ratio,* MI* myocardial infarction,* QALY* quality-adjusted life year,* RRT* renal replacement therapy,* SoC* standard of care
