# Supplementary material for: Cost-effectiveness of finerenone in chronic kidney disease associated with type 2 diabetes in The Netherlands
Source: Cardiovasc Diabetol. 2023 Nov 28;22:328. doi: 10.1186/s12933-023-02053-6 (PMC10685667; doi:10.1186/s12933-023-02053-6)
Supplement: Supplementary file 1 — Additional file 1: Incorporated standard of care. [file 12933_2023_2053_MOESM1_ESM.docx]

**Additional file 5**

**Table 1.** The use of SoC and the associated costs

| **Treatment** | **Costs per DDD^a^** | **Percentage of patients** | **Source** |
| --- | --- | --- | --- |
| **ACEIs (Enalapril, lisnopril, ramipril)** | €0.12 | 29.7% | NHG guideline [5], Guideline Diabetic Nefropathy [6], farmacotherapeutisch Kompas [34], GIP databank [32], FIDELIO-DKD [15] |
| **ARBs (Irbesartan)** | €0.21 | 59.2% | NHG guideline [5], Guideline Diabetic Nefropathy [6], farmacotherapeutisch Kompas [34], GIP databank [32], FIDELIO-DKD [15] |
| **Beta-blockers (Metropolol, bisoprolol, nebivolol)** | €0.23 | 49.6% | NHG guideline [5], Guideline Diabetic Nefropathy [6], farmacotherapeutisch Kompas [34], GIP databank [32], FIDELIO-DKD [15] |
| **Diuretics (Thiazide and lisdiuretics)** | €0.07 | 55.8% | NHG guideline [5], Guideline Diabetic Nefropathy [6], farmacotherapeutisch Kompas [34], GIP databank [32], FIDELIO-DKD [15] |
| **Calcium antagonists (Dilitazem, verapamil)** | €0.25 | 60.1% | NHG guideline [5], Guideline Diabetic Nefropathy [6], farmacotherapeutisch Kompas [34], GIP databank [32], FIDELIO-DKD [15] |
| **Statins (Simvastine, pravastatine, atorvastatine)** | €0.21 | 68.8% | NHG guideline [5], Guideline Diabetic Nefropathy [6], farmacotherapeutisch Kompas [34], GIP databank [32], FIDELIO-DKD [15] |
| **Platelet aggregation inhibitors (Aspirin)** | €0.06 | 52.2% | NHG guideline [5], Guideline Diabetic Nefropathy [6], farmacotherapeutisch Kompas [34], GIP databank [32], FIDELIO-DKD [15] |
| **Glucose-lowering therapies** | | | |
| **Insulin (insuline aspart, insuline glargine)** | €1.02 | 60.6% | NHG guideline [5], Guideline Diabetic Nefropathy [6], farmacotherapeutisch Kompas [34], GIP databank [32], FIDELIO-DKD [15] |
| **Metformin** | €0.18 | 37.6% | NHG guideline [5], Guideline Diabetic Nefropathy [6], farmacotherapeutisch Kompas [34], GIP databank [32], FIDELIO-DKD [15] |
| **Acarbose** | €0.88 | 3.7% | NHG guideline [5], Guideline Diabetic Nefropathy [6], farmacotherapeutisch Kompas [34], GIP databank [32], FIDELIO-DKD [13] |
| **Sulfonylurea (Glicazide, glimepride, tolbutamide)** | €0.15 | 20.7% | NHG guideline [5], Guideline Diabetic Nefropathy [6], farmacotherapeutisch Kompas [34], GIP databank [32], FIDELIO-DKD [15] |
| **DPP-4 inhibitors (Sitagliptine, vildagliptine, linagliptine)** | €1.74 | 26.9% | NHG guideline [5], Guideline Diabetic Nefropathy [6], farmacotherapeutisch Kompas [34], GIP databank [32], FIDELIO-DKD [15] |
| **GLP-1 agonists (Dulaglutide, liraglutide, semaglutide)** | €3.91 | 9.1% | NHG guideline [5], Guideline Diabetic Nefropathy [6], farmacotherapeutisch Kompas [34], GIP databank [32], FIDELIO-DKD [15] |
| **SGLT2 inhibitors (Canagliflozine, dapagliflozine empaglifozine,)** | €1.81 | 6.2% | NHG guideline [5], Guideline Diabetic Nefropathy [6], farmacotherapeutisch Kompas [34], GIP databank [32], FIDELIO-DKD [15] |
| ^a^ Excluding value-added tax  Abbreviations: ACE: Angiotensin-converting enzyme; ARB: Angiotensin receptor blockers; DDD: defined daily dose; DDP-4: Dipeptidyl Peptidase-4; GLP-1: glucagon-like peptide 1; SGLT2: Sodium-glucose Cotransporter-2 | | | |
