# Supplementary material for: Cost-effectiveness of finerenone in chronic kidney disease associated with type 2 diabetes in The Netherlands
Source: Cardiovasc Diabetol. 2023 Nov 28;22:328. doi: 10.1186/s12933-023-02053-6 (PMC10685667; doi:10.1186/s12933-023-02053-6)
Supplement: Supplementary file 2 — Additional file 2: Other health events rationale. [file 12933_2023_2053_MOESM2_ESM.docx]

**Additional file 1**

**Table 1.** Other Health Events rationale

| **Description** | **Identified in literature search** | **Inclusion in the model** | **Rationale** |
| --- | --- | --- | --- |
| Subsequent CV event | **✓** | **✓** | Endpoint directly related to the Main Health Events included in the model. Has an impact on costs and QALYs |
| First CV Hospitalisation (other than HF hospitalisation) | **✓** | ✘ | Inclusion of this event could lead to double counting since MI/stroke are already captured as Main Events in the model. Furthermore, there is no significant difference between arms in the FIDELIO-DKD analysis |
| Non-CV hospitalisations | ✘ | ✘ | It would be difficult to source cost and QALY data for this non-granular event. Furthermore, there are no significant differences between arms in the FIDELIO-DKD analysis. Moreover, the costs of hospital admissions non-related to CV events are included in the CKD health state costs. |
| New onset of HF | **✓** | ✘ | There is a risk of double counting with HF hospitalisation which is the composite of the Main CV Event |
| New onset of Atrial fibrillation/Atrial flutter | **✓** | **✓** | Significantly lower risk of new onset of atrial fibrillation/atrial flutter with FIN + SoC compared to SoC in FIDELIO-DKD (see in Appendix 5) and impact on costs and QALYs |
| Ear and labyrinth disorders | ✘ | ✘ | No significant differences between arms in FIDELIO-DKD |
| Eye disorders | ✘ | ✘ |  |
| Flu syndrome | ✘ | ✘ |  |
| Infections and infestations | ✘ | ✘ |  |
| Genital infections | **✓** | ✘ | Not considered in FIDELIO-DKD    Not considered in FIDELIO-DKD |
| Hypotension | **✓** | ✘ |  |
| Fractures | **✓** | ✘ |  |
| Ketoacidosis | **✓** | ✘ |  |
| Volume depletion | **✓** | ✘ |  |
| Bladder cancer | **✓** | ✘ |  |
| Gynaecomastia | **✓** | ✘ |  |
| Urosepsis | **✓** | ✘ |  |
| Amputation | **✓** | ✘ |  |
| Hypotension | **✓** | ✘ |  |
| Urinary infections | **✓** | ✘ |  |
| Pyelonephritis | **✓** | ✘ |  |
| Hyponatremia | **✓** | ✘ |  |
| Hypoglycaemia | **✓** | ✘ |  |
| Hyperkalaemia (blood potassium increased) | **✓** | **✓** | Significantly higher risk of hyperkalaemia with finerenone + SoC compared to SoC and impact on costs and QALYs |
| Sustained decrease of eGFR ≥40% from baseline | **✓ (only as a component of a composite endpoint)** | ✘ | Not considered relevant in our analysis |
| Abbreviations: CI: Confidence interval; CV: Cardiovascular; eGFR: estimated glomerular filtration rate; HF: Heart failure; HR: Hazard ratio; SoC: Standard of Care; QALY: Quality-adjusted life year | | | |
