# Supplementary material for: Cost-effectiveness of finerenone in chronic kidney disease associated with type 2 diabetes in The Netherlands
Source: Cardiovasc Diabetol. 2023 Nov 28;22:328. doi: 10.1186/s12933-023-02053-6 (PMC10685667; doi:10.1186/s12933-023-02053-6)
Supplement: Supplementary file 3 — Additional file 3: Description of the calculation of transition probabilities for CKD progression and CV events, and corresponding input data. [file 12933_2023_2053_MOESM3_ESM.docx]

**Additional file 2**

Description of Transition probabilities for CKD progression and first modelled CV event

In the FIDELIO-DKD trial, at every four-month interval (i.e., the model cycle length), patients were observed as belonging to one of the CKD health stages which correspond to the CKD health states in the model [13]. The transition probabilities for both arms (SoC and finerenone in combination with SoC) used in the model were calculated as the average probabilities over the four years available from the FIDELIO-DKD trial [13].

Additional analyses of the FIDELIO-DKD patient-level data provided estimates of the risk of first-modelled CV events at different points in time (e.g., events in each four-month interval) for patients in each CKD and ESKD stage. The risk of the first modelled CV event (used in the model) was calculated as an average rate over the four years of data available from the FIDELIO-DKD trial analysis [13]. An average CV event was defined as a result of a lack of reliable data to calculate the required transitions and to avoid overly complex model programming. The definition of this average CV event was based on events included in the cardiovascular composite endpoint: non-fatal MI, non-fatal stroke, and hospitalization for heart failure. It was assumed that CV risk increases with age. Therefore, to extrapolate the CV probabilities to a lifetime horizon, an HR of 1.03 (95%CI 1.03–1.04) for increased CV risk was used after four years. The HR was based on increased age from Wilson 2012 and was applied to the baseline risks in the SoC arm of the model from the FIDELIO-DKD analysis [58]. Table A1 shows the distribution of CV events used to assess the impact of CV events on costs and utilities.

**Table 1.** Distribution of CV events

| **Description** | **MI** | **IS stroke** | **ICH stroke** | **HF hospitalization** |
| --- | --- | --- | --- | --- |
| % of patients who experienced an event | 20.7% | 21.0% | 2.0% | 56.3% |
| Abbreviations: HF: Heart failure; IS: Ischemic; ICH: intracerebral brain haemorrhage; MI: Myocardial infarct | | | | |
