# Supplementary material for: Cost-effectiveness of finerenone in chronic kidney disease associated with type 2 diabetes in The Netherlands
Source: Cardiovasc Diabetol. 2023 Nov 28;22:328. doi: 10.1186/s12933-023-02053-6 (PMC10685667; doi:10.1186/s12933-023-02053-6)
Supplement: Supplementary file 4 — Additional file 4: Description of the calculation of mortality and corresponding input data. [file 12933_2023_2053_MOESM4_ESM.docx]

**Additional file 3**

Description of the calculation of mortality

The reasons for death considered in the model comprised renal death, CV death, and mortality from other causes. The average risk of CV death was retrieved from the FIDELIO-DKD analysis and implemented for each cycle in the model for patients without CV events [14]. The average risk of renal death was based on the FIDELIO-DKD analysis and was implemented in the case of patients with eGFR < 15 mL/min/1.73 m2 (before dialysis or a kidney transplant) [14]. In addition to renal and CV deaths, mortality from other causes which was considered in the model, was based on Dutch statistical data [22]. Both CV and CKD deaths were subtracted from the background mortality to avoid double-counting.

Mortality increased with (long-term) CKD progression, which was not captured in the FIDELIO-DKD analysis. HRs associated with each of the modelled health states were applied to the model based on Darlington 2021 (Table A2) [20]. The increased mortality rates for patients with dialysis or kidney transplants were based on the UKRR report [21]. Increased mortality following the first modelled CV event was also considered in the model. The HRs based on the cost-effectiveness analysis by Erickson 2013 were applied for patients in each cycle post-CV event (Table A2) [19]. Erickson 2013 presented the long-term increase in mortality after MI and stroke for patients who survived the acute event. The four-month probability for CV and renal death is presented in Table A3. Table A4 illustrates the mortality from other causes (with renal and CV death subtracted).

**Table 1.** HRs used to adjust for an increased mortality risk in patients

| Description | HR | Reference |
| --- | --- | --- |
| CKD 1/2 | 1.14 (0.96-1.15) | [20]  Calculated as the average of HRs for CKD 1 and CKD 2. Weighting with FIDELIO-DKD data not possible - % of patients in CKD 1 unknown |
| CKD 3 | 1.33 (1.23-1.40) | [20]  Calculated as the average of HRs for CKD 3A and 3B and weighted by the % of CKD 3A and 3B patients from FIDELIO-DKD |
| CKD 4 | 6.42 (5.19-9.50) | [20] |
| CKD 5 w/o RRT | 9.49 (9.49-9.49) | [20] |
| Dialysis, acute | 10.04 (5.39-13.23) | [21] |
| Dialysis, post-acute | 10.04 (5.39-13.23) | [21] |
| Transplant, acute | 1.55 (0.83-2.04) | [21] |
| Transplant, post-acute | 1.55 (0.83-2.04) | [21] |
| MI | 1.40 (1.07-2.04) | [19] |
| Stroke | 2.30 (2.00-2.70) | [19] |
| Heart failure | 1.40 (1.07-2.40) | [19] |
| Abbreviations: CKD: Chronic kidney disease; HR: Hazard ratio; MI: Myocardial Infarct; RRT: Renal replacement therapy | | |

**Table 2**. **Mortality from other causes (not CV and renal related)**

| **Age (years)** | **Annual mortality risk (males)** | **Annual mortality risk (females)** |
| --- | --- | --- |
| 65 | 1.08% | 0.75% |
| 66 | 1.23% | 0.83% |
| 67 | 1.34% | 0.91% |
| 68 | 1.47% | 1.02% |
| 69 | 1.63% | 1.11% |
| 70 | 1.74% | 1.17% |
| 71 | 2.04% | 1.33% |
| 72 | 2.21% | 1.47% |
| 73 | 2.45% | 1.60% |
| 74 | 2.63% | 1.74% |
| 75 | 3.20% | 2.05% |
| 76 | 3.56% | 2.35% |
| 77 | 3.92% | 2.53% |
| 78 | 4.47% | 2.91% |
| 79 | 5.02% | 3.32% |
| 80 | 5.61% | 3.61% |
| 81 | 6.36% | 4.26% |
| 82 | 6.91% | 4.78% |
| 83 | 8.19% | 5.44% |
| 84 | 9.27% | 6.34% |
| 85 | 10.07% | 7.03% |
| 86 | 11.69% | 8.49% |
| 87 | 13.54% | 9.87% |
| 88 | 14.95% | 11.19% |
| 89 | 17.36% | 12.59% |
| 90 | 19.15% | 14.87% |
| 91 | 20.77% | 16.36% |
| 92 | 23.07% | 18.31% |
| 93 | 24.62% | 20.46% |
| 94 | 27.89% | 22.73% |
| 95 | 31.11% | 24.34% |
| 96 | 31.20% | 27.43% |
| 97 | 35.14% | 30.57% |
| 98 | 38.68% | 31.72% |
| 99 | 40.20% | 38.23% |
| 100 | 40.20% | 38.23% |

**Table 3**. The proportion of death caused by CV events and CKD

|  | **CV deaths** | | **CKD deaths** | |
| --- | --- | --- | --- | --- |
| **Age range** | Males | Females | Males | Females |
| 15-19 years | 5.79% | 4.23% | 0.00% | 0.00% |
| 20-24 years | 3.00% | 0.00% | 0.00% | 0.00% |
| 25-29 years | 3.70% | 4.62% | 0.00% | 0.77% |
| 30-34 years | 5.73% | 7.29% | 0.00% | 0.52% |
| 35-39 years | 9.91% | 12.26% | 0.00% | 0.00% |
| 40-44 years | 12.41% | 9.79% | 0.00% | 0.23% |
| 45-49 years | 16.70% | 13.64% | 0.18% | 0.00% |
| 50-54 years | 18.80% | 11.15% | 0.19% | 0.29% |
| 55-59 years | 18.10% | 10.35% | 0.33% | 0.19% |
| 60-64 years | 17.89% | 11.12% | 0.19% | 0.38% |
| 65-69 years | 19.08% | 13.60% | 0.26% | 0.41% |
| 70-74 years | 20.59% | 16.27% | 0.46% | 0.40% |
| 75-79 years | 20.92% | 19.58% | 0.47% | 0.57% |
| 80-84 years | 22.94% | 22.31% | 0.70% | 0.56% |
| 85-89 years | 24.59% | 25.86% | 0.94% | 0.65% |
| 90+ years | 26.49% | 28.01% | 0.90% | 0.61% |
