# Supplementary material for: Cost-effectiveness of finerenone in chronic kidney disease associated with type 2 diabetes in The Netherlands
Source: Cardiovasc Diabetol. 2023 Nov 28;22:328. doi: 10.1186/s12933-023-02053-6 (PMC10685667; doi:10.1186/s12933-023-02053-6)
Supplement: Supplementary file 5 — Additional file 5: Utility values incorporated in the base case and scenario analyses. [file 12933_2023_2053_MOESM5_ESM.docx]

**Additional file 4**

**Table 1.** Utility used in base case

| **Parameter** | **Utility value** | | **Source** |
| --- | --- | --- | --- |
| Baseline utility value | 0.804 | | Retrieved from the FIDELIO-DKD trial and calculated with the Dutch EQ-5D-5L value set [13,23] |
| CKD 1/2 | 0.804 | |  |
| CKD 3 | 0.804 | |  |
| CKD 4 | 0.793 | |  |
| CKD 5 w/o RRT | 0.772 | |  |
| Dialysis | 0.463 | | Tolvaptan (TA358) [24] |
| Post-dialysis | 0.463 | | Tolvaptan (TA358) [24] |
| Transplant | 0.656 | | Tolvaptan (TA358) [24] |
| Post-transplant | 0.722 | | Tolvaptan (TA358) [24] |
|  | **Acute** | **Post-acute** |  |
| MI | -0.039 | -0.039 | Retrieved from the FIDELIO-DKD trial and calculated with the Dutch EQ-5D-5L value set [13,23] |
| Stroke | -0.054 | -0.054 |  |
| Hospitalisation for HF | -0.042 | -0.042 |  |
| New onset of Atrial fibrillation/ Atrial flutter | 0.000 | |  |
| Hyperkalaemia leading to hospitalisation | -0.005 | |  |
| Hyperkalaemia not leading to hospitalisation | -0.005 | |  |
| Subsequent CV event | -0.044 | |  |
| Abbreviations: CKD: Chronic kidney disease; RRT: Renal replacement therapy | | | |

**Table 2.** Utility based on trial (scenario analysis)

| **Parameter** | **Utility value** | | **Source** |
| --- | --- | --- | --- |
| Baseline utility value |  | | Retrieved from the FIDELIO-DKD trial and calculated with the Dutch EQ-5D-5L value set [13,23] |
| CKD 1/2 | 0.804 | |  |
| CKD 3 | 0.804 | |  |
| CKD 4 | 0.793 | |  |
| CKD 5 w/o RRT | 0.772 | |  |
| Dialysis | 0.740 | |  |
| Post-dialysis | 0.736 | |  |
| Transplant | 0.791 | |  |
| Post-transplant | 0.888 | |  |
|  | **Acute** | **Post-acute** |  |
| MI | -0.039 | -0.039 | Retrieved from the FIDELIO-DKD trial and calculated with the Dutch EQ-5D-5L value set [13,23] |
| Stroke | -0.054 | -0.054 |  |
| Hospitalisation for HF | -0.042 | -0.042 |  |
| New onset of Atrial fibrillation/ Atrial flutter | 0.000 | |  |
| Hyperkalaemia leading to hospitalisation | -0.005 | |  |
| Hyperkalaemia not leading to hospitalisation | -0.005 | |  |
| Subsequent CV event | -0.044 | |  |
| Abbreviations: CKD: Chronic kidney disease; RRT: Renal replacement therapy | | | |

**Table 3.** Utility based on data retrieved in the systematic literature review (scenario analysis)

| **Parameter** | **Utility value** | | **Source** |
| --- | --- | --- | --- |
| Baseline utility value | 0.804 | | Retrieved from the FIDELIO-DKD trial and calculated with the Dutch EQ-5D-5L value set [13,23] |
| CKD 1/2 | 0.774 | | Tolvaptan (TA358) [24] |
| CKD 3 | 0.754 | | Tolvaptan (TA358) [24] |
| CKD 4 | 0.582 | | Tolvaptan (TA358) [24] |
| CKD 5 w/o RRT | 0.463 | | Tolvaptan (TA358) [24] |
| Dialysis | 0.463 | | Tolvaptan (TA358) [24] |
| Post-dialysis | 0.656 | | Tolvaptan (TA358) [24] |
| Transplant | 0.656 | | Tolvaptan (TA358) [24] |
| Post-transplant | 0.722 | | Tolvaptan (TA358) [24] |
|  | **Acute** | **Post-acute** |  |
| MI | -0.139 | -0.070 | Meads et al.[61] |
| Stroke | -0.160 | -0.080 | Meads et al.[61] |
| Hospitalisation for HF | -0.321 | -0.025 | McEwan et al.[44] |
| New onset of Atrial fibrillation/ Atrial flutter | -0.014 | | Rincoig et al. [62] |
| Hyperkalaemia leading to hospitalisation | -0.030 | | Palaka et al.[63] |
| Hyperkalaemia not leading to hospitalisation | -0.030 | | Palaka et al. [63] |
| Subsequent CV event | -0.246 | | Meads et al.[61] |
| Abbreviations: CKD: Chronic kidney disease; RRT: Renal replacement therapy | | | |

**Table 4.** Utility based on data retrieved from the literature validated by the Dutch Healthcare Institute

| **Parameter** | **Utility value** | | **Source** |
| --- | --- | --- | --- |
| Baseline utility value | 0.804 | | Retrieved from the FIDELIO-DKD trial and calculated with the Dutch EQ-5D-5L value set [13,23] |
| CKD 1/2 | 0.804 | | Jesky et al [64] |
| CKD 3 | 0.804 | | Jesky et al [64] |
| CKD 4 | 0.793 | | Jesky et al [64] |
| CKD 5 w/o RRT | 0.772 | | Jesky et al [64] |
| Dialysis | 0.540 | | Lee et al [65] |
| Post-dialysis | 0.540 | | Lee et al [65] |
| Transplant | 0.740 | | Lee et al [65] |
| Post-transplant | 0.740 | | Lee et al [65] |
|  | **Acute** | **Post-acute** |  |
| MI | -0.091 | -0.070 | Briggs et al [66] |
| Stroke | -0.107 | -0.080 | Briggs et al [66] |
| Hospitalisation for HF | -0.074 | -0.025 | Briggs et al [66] |
| New onset of Atrial fibrillation/ Atrial flutter | -0.014 | | Rincoig et al [62] |
| Hyperkalaemia leading to hospitalisation | -0.030 | | Palaka et al [63] |
| Hyperkalaemia not leading to hospitalisation | -0.030 | | Palaka et al [63] |
| Subsequent CV event | -0.085 | | Briggs et al [66] |
| Abbreviations: CKD: Chronic kidney disease; RRT: Renal replacement therapy | | | |
