# Supplementary material for: Cost-effectiveness of finerenone in chronic kidney disease associated with type 2 diabetes in The Netherlands
Source: Cardiovasc Diabetol. 2023 Nov 28;22:328. doi: 10.1186/s12933-023-02053-6 (PMC10685667; doi:10.1186/s12933-023-02053-6)
Supplement: Supplementary file 6 — Additional file 6: Costs and resource allocation used to calculate the costs per CKD health state. [file 12933_2023_2053_MOESM6_ESM.docx]

**Additional file 6**

**Table 1.** Cost inputs and resource allocation of the CKD health states

| **Parameter** | **Value** | **Source** |
| --- | --- | --- |
| **Cost inputs (not adjusted for inflation)** | | |
| Control visit GP | €30 | Dutch costing manual [35] |
| Outpatient visit | €91 | Dutch costing manual [35] |
| eGFR measurement | €6.14 | NZA: 070128, 070442, 070443) [37] |
| Albuminuria measurement | €4.55 | NZA: 071739 [37] |
| Treatment with an ACE-inhibitor | €0.13 per day | farmacotherapeutisch Kompas (36), GIP databank (37), |
| Influenza vaccination | €20.33 | De Boer et al [64] |
| Hospital admission | €476 | Dutch costing manual [35] |
| **Resource utilization per cycle per CKD progression health state** | | |
| CKD 1/2 | Control visit GP: 0.33  eGFR measurement (natrium, kalium, creatine): 0.33  Albuminuria measurement: 0.33  Treatment with ACE-inhibitor: No  Hospital admission (unrelated to CV outcomes): 0.09 | NHG guideline [5] Schrauben et al [36] |
| CKD 3a/b | Control visit GP: 0.66  eGFR measurement (natrium, kalium, creatine): 0.66  Albuminuria measurement: 0.66  Treatment with ACE-inhibitor: Yes  Influenza vaccination: Yes  Hospital admission (unrelated to CV outcomes): 0.114 | NHG guideline [5] Schrauben et al [36] |
| CKD 4 | Visit GP: 0.66  Outpatient visit: 1.3  eGFR measurement (natrium, kalium, creatine): 1.3  Albuminuria measurement: 1.3  Treatment with ACE-inhibitor: Yes  Influenza vaccination: Yes  Hospital admission (unrelated to CV outcomes): 0.124 | NHG guideline [5] Schrauben et al [36] |
| CKD 5 (without RRT) | Outpatient visit: Every 6 weeks  eGFR measurement (natrium, kalium, creatine): Every 6 weeks  Albuminuria measurement: Every 6 weeks  Treatment with ACE-inhibitor: Yes  Influenza vaccination: Yes  Hospital admission (unrelated to CV outcomes): 0.124 | NHG guideline [5] Schrauben et al [36] |
| Abbreviations: : ACE: Angiotensin-converting enzyme; CV: Cardiovascular; eGFR: estimated glomerular filtration rate; GP: General practitioner; RRT: Renal replacement therapy | | |
