# Supplementary material for: Cost-effectiveness of finerenone in chronic kidney disease associated with type 2 diabetes in The Netherlands
Source: Cardiovasc Diabetol. 2023 Nov 28;22:328. doi: 10.1186/s12933-023-02053-6 (PMC10685667; doi:10.1186/s12933-023-02053-6)
Supplement: Supplementary file 7 — Additional file 7: Healthcare claims used to calculate the costs related to dialysis and transplantation. [file 12933_2023_2053_MOESM7_ESM.docx]

**Additional file 7**

**Table 1.** Overview of included dialysis costs based on Dutch healthcare claims (corrected for 2022 inflation)

|  | **Weighted average haemodialysis** | **Weighted average peritoneal dialysis** | **Source** |
| --- | --- | --- | --- |
| **Direct costs related to dialysis** | | | Mohnen et al. [28] |
| Dialysis modality | € 87,898 | €85,345 |  |
| Dialysis access | € 2,039 | €558 |  |
| **Indirect costs related to dialysis** | | |  |
| Hospital | €10,558 | €10,342 |  |
| Primary care | €493 | €437 |  |
| Mental health care | €283 | €149 |  |
| Medication related to dialysis | € 5,431 | €5,276 |  |
| Medical devices | €1,260 | €2,364 |  |
| Healthcare abroad | €226 | €150 |  |
| Transportation | €686 | €601 |  |
| Other | €993 | €854.66 |  |
| **Total annual costs** | **€109,866** | **€ 106,077** |  |

**Table 2.** Overview of included transplantation costs based on Dutch healthcare claims (corrected for 2022 inflation)

|  | **Year of transplantation** | **>Second year after successful transplantation** | **Source** |
| --- | --- | --- | --- |
| **Direct costs related to transplantation** | | | Mohnen et al. [28] |
| Preparatory research | €2,455 | €24 |  |
| Transplant operation | €28,580 | €69 |  |
| Guidance | €596 | €136 |  |
| After care | €8,547 | €2,283 |  |
| Doner expenses | €3,277 | €6 |  |
| **Indirect costs related to transplantation** | | |  |
| Hospital | €11,999 | €5,897 |  |
| Primary care | €309 | €353 |  |
| Mental health care | €185 | €256 |  |
| Medication related to transplantation | €11,593 | €8,276 |  |
| Medical devices | €852 | €814 |  |
| Health care abroad | €270 | €65 |  |
| Transportation | €2,811 | € 431 |  |
| Other | €276 | €226 |  |
| **Total annual costs** | **€71,749** | **€16,319** |  |
