# Supplementary material for: Cost-effectiveness of finerenone in chronic kidney disease associated with type 2 diabetes in The Netherlands
Source: Cardiovasc Diabetol. 2023 Nov 28;22:328. doi: 10.1186/s12933-023-02053-6 (PMC10685667; doi:10.1186/s12933-023-02053-6)
Supplement: Supplementary file 9 — Additional file 9: Overview of included parameters and their distributions that was used in the probabilistic sensitivity analysis. [file 12933_2023_2053_MOESM9_ESM.docx]

**Additional file 10**

**Table 1.** Overview of included parameters and their distributions

| **Parameter** | **Deterministic value (97.5% CI)** | **Distribution** | **Reference** |
| --- | --- | --- | --- |
| Mean age | 65.6 (47.8 – 83.4) | Normal | FIDELIO-DKD [13] |
| Proportion of males | 70.2% (69.0-71.4%) | Beta | FIDELIO-DKD [13] |
| Baseline patients distribution (CKD1/2, CKD3, CKD4, CKD5 w/o RRT, Dialysis, Kidney Transplant) | 11.6, 76.2, 12.3 | Dirichlet | FIDELIO-DKD [13] |
| Cumulative risk of premature discontinuation at 4 years, finerenone | 0.395 (0.366-0.424) | Beta | FIDELIO-DKD [13] |
| Four-month risk of first modelled CV event, CKD1/2 | 0.012 (0.008-0.017) | Beta | FIDELIO-DKD [13] |
| Four-month risk of first modelled CV event, CKD3 | 0.013 (0.011-0.015) | Beta | FIDELIO-DKD [13] |
| Four-month risk of first modelled CV event, CKD4 | 0.016 (0.012-0.019) | Beta | FIDELIO-DKD [13] |
| Four-month risk of first modelled CV event, CKD 5 w/o RRT | 0.021 (0.008-0.039) | Beta | FIDELIO-DKD [13] |
| Four-month risk of first modelled CV event, Dialysis (acute) | 0.021 (0.008-0.039) | Beta | FIDELIO-DKD [13] |
| Four-month risk of first modelled CV event, Dialysis (post-acute) | 0.021 (0.008-0.039) | Beta | FIDELIO-DKD [13] |
| Four-month risk of first modelled CV event, Kidney Transplant (acute) | 0.016 (0.012-0.019) | Beta | FIDELIO-DKD [13] |
| Four-month risk of first modelled CV event, Kidney Transplant (post-acute) | 0.016 (0.012-0.019) | Beta | FIDELIO-DKD [13] |
| Increased risk of first modelled CV event, HR due to age | 1.03 (1.03-1.04) | LogNormal | Wilson et al. [57] |
| Time after which risk of first modelled CV event is increased | 4.0 (3.6-4.4) | LogNormal | Assumption |
| Four-month risk of hyperkalaemia leading to hospitalisation, no-CV event | 0.001 (0.000-0.001) | Beta | FIDELIO-DKD [13] |
| Four-month risk of new onset of atrial fibrillation / atrial flutter, no-CV event | 0.004 (0.003-0.004) | Beta | FIDELIO-DKD [13] |
| Four-month risk of hyperkalaemia not-leading to hospitalisation, no-CV event | 0.016 (0.015-0.018) | Beta | FIDELIO-DKD [13] |
| Four-month risk of hyperkalaemia, post-CV event | 0.073 (0.061-0.087) | Beta | FIDELIO-DKD [13] |
| Four-month risk of new onset of atrial fibrillation / atrial flutter, post-CV event | 0.022 (0.015-0.029) | Beta | FIDELIO-DKD [13] |
| Four-month risk of hyperkalaemia not-leading to hospitalisation, post-CV event | 0.024 (0.017-0.032) | Beta | FIDELIO-DKD [13] |
| Duration of hyperkalaemia leading to hospitalisation | 0.33 (0.14-0.67) | Beta | Assumption |
| Duration of atrial fibrillation/atrial flutter | 0.33 (0.14-0.67) | Beta | Assumption |
| Duration of hyperkalaemia not leading to hospitalisation | 0.33 (0.14-0.67) | Beta | Assumption |
| Four-month CV death probability, CKD1/2 | 0.006 (0.003-0.010) | Beta | FIDELIO-DKD |
| Four-month CV death probability, CKD3 | 0.005 (0.004-0.006) | Beta | FIDELIO-DKD |
| Four-month CV death probability, CKD4 | 0.008 (0.006-0.011) | Beta | FIDELIO-DKD |
| Four-month CV death probability, CKD5 w/o RRT | 0.016 (0.006-0.030) | Beta | FIDELIO-DKD |
| Four-month CV death probability, Dialysis (acute) | 0.019 (0.006-0.039) | Beta | FIDELIO-DKD |
| Four-month CV death probability, Dialysis (post-acute) | 0.019 (0.006-0.039) | Beta | FIDELIO-DKD |
| Four-month CV death probability, Kidney Transplant (acute) | 0.008 (0.006.0.011) | Beta | FIDELIO-DKD |
| Four-month CV death probability, Kidney Transplant (post-acute) | 0.008 (0.006-0.011) | Beta | FIDELIO-DKD |
| Four-month renal death probability, CKD5 w/o RRT | 0.0000 (0.0000-0.0000) | Beta | FIDELIO-DKD |
| Increased mortality risk, HR due to CKD1/2 | 1.14 (1.04-1.24) | LogNormal | Darlington et al. [20] |
| Increased mortality risk, HR due to CKD3 | 1.33 (1.25-1.42) | LogNormal | Darlington et al. [20] |
| Increased mortality risk, HR due to CKD4 | 6.42(4.75-8.69) | LogNormal | Darlington et al. [20] |
| Increased mortality risk, HR due to CKD5 w/o RRT | 9.49 (6.89-13.07) | LogNormal | Darlington et al. [20] |
| Increased mortality risk, HR due to Dialysis (acute) | 10.04 (6.41-15.73) | LogNormal | UKKR report [21] |
| Increased mortality risk, HR due to Dialysis (post-acute) | 10.04 (6.41-15.73) | LogNormal | UKKR report [21] |
| Increased mortality risk, HR due to Kidney Transplant (acute) | 1.55 (0.99-2.43) | LogNormal | UKKR report [21] |
| Increased mortality risk, HR due to Kidney Transplant (post-acute) | 1.55 (0.99-2.43) | LogNormal | UKKR report [21] |
| Increased mortality risk, HR due to first MI | 1.40 (1.01-1.93) | LogNormal | Erickson et al. [19] |
| Increased mortality risk, HR due to first stroke | 2.30 (1.98-2.67) | LogNormal | Erickson et al. [19] |
| Increased mortality risk, HR due to first hospitalisation for HF | 1.40 (1.01-1.93) | LogNormal | Erickson et al. [19] |
| HR: Onset of eGFR decrease < 15 mL/min, FIN+BT vs BT | 0.82 (0.67-1.01) | LogNormal | FIDELIO-DKD [13] |
| HR: Progression to dialysis, FIN + SoC vs SoC | 0.87 (0.67-1.12) | LogNormal | FIDELIO-DKD [13] |
| HR: Progression to kidney transplant Fin + SoC vs SoC | 1.00 (1.00-1.00) | N/A | FIDELIO-DKD [13] |
| HR: CV death, FIN + SOC vs SOC | 0.86 (0.68-1.08) | LogNormal | FIDELIO-DKD [13] |
| HR: Renal death, CKD 5 w/o RRT, FIN + SoC vs SoC | 1.03 (0.14-7.30) | LogNormal | FIDELIO-DKD [13] |
| HR: First modelled CV event, FIN + SoC vs SoC | 0.87 (0.74-1.02) | LogNormal | FIDELIO-DKD [13] |
| HR: Subsequent CV event, FIN + SoC vs SoC | 0.95 (0.70-1.30) | LogNormal | FIDELIO-DKD [13] |
| HR: Hyperkalaemia leading to hospitalisation, FIN + SoC vs SoC | 2.71 (1.60-4.60) | LogNormal | FIDELIO-DKD [13] |
| HR: New onset of atrial fibrillation / atrial flutter, FIN + SoC vs SoC | 0.71 (0.53-0.94) | LogNormal | FIDELIO-DKD [13] |
| HR: Hyperkalaemia not-leading to hospitalisation, FIN + SOC vs SOC | 1.92 (1.67-2.21) | LogNormal | FIDELIO-DKD [13] |
| % of patients who use a 10 mg dose of finerenone | 34.8% (34.1-35.7) | Beta | FIDELIO-DKD [13] |
| % of patients who use ACEIs | 29.7% (28.5-30.9) | Beta | FIDELIO-DKD [13] |
| % of patients who use ARBs | 59.2% (57.9-60.4) | Beta | FIDELIO-DKD [13] |
| % of patients who use beta-blockers | 49.6% (48.3-50.9) | Beta | FIDELIO-DKD [13] |
| % of patients who use diuretics | 55.8% (54.4-57.1) | Beta | FIDELIO-DKD [13] |
| % of patients who use calcium antagonists | 60.1% (58.8-61.4) | Beta | FIDELIO-DKD [13] |
| % of patients who use statins | 68.8% (67.6-70.0) | Beta | FIDELIO-DKD [13] |
| % of patients who use platelet aggregation inhibitors | 52.2% (50.9-53.5) | Beta | FIDELIO-DKD [13] |
| % of patients who use insulin | 60.6% (59.3-61.9) | Beta | FIDELIO-DKD [13] |
| % of patients who use metformin | 37.6% (36.3-38.9) | Beta | FIDELIO-DKD [13] |
| % of patients who use acarbose | 3.7% (3.3-4.2) | Beta | FIDELIO-DKD [13] |
| % of patients who use sulfonylurea | 20.7% (19.7-21.8) | Beta | FIDELIO-DKD [13] |
| % of patients who use DPP-4 inhibitors | 26.9% (25.7-28.0) | Beta | FIDELIO-DKD [13] |
| % of patients who use GLP-1 agonists | 9.1% (8.3-9.8) | Beta | FIDELIO-DKD [13] |
| % of patients who use SGLT2 inhibitors | 6.2% (5.6-6.8) | Beta | FIDELIO-DKD [13] |
| Daily cost of ACEIs | 0.12 (0.07-0.19) | Gamma | FIDELIO-DKD [13] |
| Daily cost of ARBs | 0.21 (0.12-0.32) | Gamma | FIDELIO-DKD [13] |
| Daily cost of beta-blockers | 0.23 (0.13-0.36) | Gamma | FIDELIO-DKD [13] |
| Daily cost of diuretics | 0.07 (0.04-0.11) | Gamma | FIDELIO-DKD [13] |
| Daily cost of calcium antagonists | 0.25 (0.14-0.39) | Gamma | FIDELIO-DKD [13] |
| Daily cost of statins | 0.21 (0.12-0.32) | Gamma | FIDELIO-DKD [13] |
| Daily cost of platelet aggregation inhibitors | 0.06 (0.04-0.10) | Gamma | FIDELIO-DKD [13] |
| Daily cost of insulin | 1.02 (0.58-1.58) | Gamma | FIDELIO-DKD [13] |
| Daily cost of metformin | 0.18 (0.10-0.28) | Gamma | FIDELIO-DKD [13] |
| Daily cost of acarbose | 0.88 (0.50-1.36) | Gamma | FIDELIO-DKD [13] |
| Daily cost of sulfonylurea | 0.15 (0.09-0.24) | Gamma | FIDELIO-DKD [13] |
| Daily cost of DPP-4 inhibitors | 1.74 (0.99-2.69) | Gamma | FIDELIO-DKD [13] |
| Daily cost of GLP-1 agonists | 3.91 (2.24-6.05) | Gamma | FIDELIO-DKD [13] |
| Daily cost of SGLT2 inhibitors | 1.81 (1.03-2.79) | Gamma | FIDELIO-DKD [13] |
| CKD1/2 management cost per cycle | 74 (42-114) | Gamma | FIDELIO-DKD [13] |
| CKD3 management cost per cycle | 140 (80-216) | Gamma | FIDELIO-DKD [13] |
| CKD4 management cost per cycle | 303 (173-469) | Gamma | FIDELIO-DKD [13] |
| CKD 5 w/o RRT management cost per cycle | 467 (267-722) | Gamma | FIDELIO-DKD [13] |
| % of haemodialysis in all dialysis | 88.2 (77.6-82.3) | Beta | FIDELIO-DKD [13] |
| Cost of haemodialysis (acute), per cycle | 39,568 (22,617-61,183) | Gamma | Mohnen et al. [28] |
| Cost of haemodialysis (post-acute), per cycle | 39,568 (22,617-61,183) | Gamma | Mohnen et al. [28] |
| Cost of peritoneal dialysis (acute), per cycle | 36,256 (20,723-56,061-) | Gamma | Mohnen et al. [28] |
| Cost of peritoneal dialysis (post-acute), per cycle | 36,256 (20,723-56,061-) | Gamma | Mohnen et al. [28] |
| Cost of kidney transplant (acute, year of transplantation), per cycle | 24,523 (14,017-37,919) | Gamma | Mohnen et al. [28] |
| Cost of kidney transplant (post-acute), per cycle | 6,449 (3,686-9,972) | Gamma | Mohnen et al. [28] |
| First modelled CV events distribution (MI, IS stroke, ICH stroke, Hospitalization for HF) | 20.7% (2-56.3) | Dirichlet | FIDELIO-DKD [13] |
| Cost of MI (acute) | 4,038 (2,308-6,244) | Gamma | Van Schoonhoven et al. [29] |
| Cost of MI (post-acute) | 748 (428-1,157) | Gamma | Van Schoonhoven et al. [29] |
| Cost of IS stroke (acute) | 11,169 (6,384-17,270) | Gamma | Van Schoonhoven et al. [29] |
| Cost of IS stroke (post-acute) | 3,157 (1,805-4,822) | Gamma | Van Schoonhoven et al. [29] |
| Cost of ICH stroke (acute) | 11,169 (6,384-17,270) | Gamma | Van Schoonhoven et al. [29] |
| Cost of ICH stroke (post-acute) | 3,157 (1,805-4,822) | Gamma | Van Schoonhoven et al. [29] |
| Cost of hospitalization for HF (acute) | 2,578 (1,473-3,986) | Gamma | Van Schoonhoven et al. [29] |
| Cost of hospitalization for HF (post-acute) | 274 (157-424) | Gamma | Van Schoonhoven et al. [29] |
| Cost of hyperkalaemia leading to hospitalisation | 2,873 (1,642-4,442) | Gamma | GIP databank [32] |
| Cost of new onset of atrial fibrillation / atrial flutter | 1,160 (663-1,793) | Gamma | Ringborg et al. [31] |
| Cost of hyperkalaemia not-leading to hospitalisation | 306 (175-472) | Gamma | Dutch costing manual [35] |
| Percentage of patients working CKD 1/2 | 69% (4.0-100%) | Beta | CBS [42] |
| Percentage of patients working CKD 3 to5 | 68% (3.9-100%) | Beta | Alma et al [40] |
| Percentage of patients working dialysis | 52% (1.6-100%) | Beta | Alma et al [40] |
| Percentage of patients working transplantation | 64% (3.2-100%) | Beta | Alma et al [40] |
| Gross hourly wage patient | 30 (17-46) | Gamma | CBS Statline [35] |
| Opportunity costs caretaker | 18 (10-28) | Gamma | Dutch costing manual [35] |
| Number of days of informal care MI | 4 (2-6) | Gamma | Kotseva et al [38] Expert opinion |
| Number of days of informal care after stroke | 4 (2-6) | Gamma | Kotseva et al [38]  Expert opinion |
| Number of days of informal care after HF | 4 (2-6) | Gamma | Kotseva et al [38]  Expert opinion |
| Number of days of informal care after dialysis | 15 (12-23) | Gamma | Kotseva et al [38]  Expert opinion |
| Number of days of informal care after transplantation | 15 (12-24) | Gamma | Kotseva et al [38]  Expert opinion |
| Number of days of productivity losses MI | 20 (11-30) | Gamma | Kotseva et al [38]  Expert opinion |
| Number of days of productivity losses stroke | 19 (11-29) | Gamma | Kotseva et al [38]  Expert opinion |
| Number of days of productivity losses after HF | 20 (11-30) | Gamma | Kotseva et al [38]  Expert opinion |
| Number of days of productivity losses after dialysis | 13 (7-20) | Gamma | Alma et al. [40] |
| Number of days of productivity losses transplantation | 7 (4-11) | Gamma | Alma et al. [40] |
| Number of days of informal care MI (post-acute) | 0 (0-0) | Gamma | Kotseva et al [38]  Expert opinion |
| Number of days of informal care after stroke (post-acute) | 1 (0-1) | Gamma | Kotseva et al [38]  Expert opinion |
| Number of days of informal care after HF (post-acute) | 1 (0-1) | Gamma | Kotseva et al [38]  Expert opinion |
| Number of days of informal care after transplantation (post-acute) | 0 | Gamma | De Vries et al. [39] |
| Number of days of productivity losses after MI (post-acute) | 1 (0-1) | Gamma | De Vries et al. [39] |
| Number of days of productivity losses after stroke (post-acute) | 3 (2-5) | Gamma | De Vries et al. [39] |
| Number of days of productivity losses after HF (post-acute) | 3 (2-5) | Gamma | De Vries et al. [39] |
| Number of days of productivity losses after transplant (post-acute) | 4 (4-7) | Gamma | De Vries et al. [39] |
| Number of days of productivity losses after dialysis (post-acute) | 5 (4-8) | Gamma | De Vries et al. [39] |
| CKD 3-5- productivity losses (including sick leave) | 7 (4-12) | Gamma | Alma et al. [40] |
| CKD 3-5- productivity losses (excluding sick leave) | 4 (3-7) | Gamma | Alma et al. [40] |
| CKD 4 informal care | 4 (4-7) | Gamma | Alma et al. [40] |
| CKD 5 informal care | 5 (4-7) | Gamma | Alma et al. [40] |
| CKD1/2 utility | 0.804 (0.274-0.997) | Beta | FIDELIO-DKD [13]  Versteegh et al. [23] |
| CKD3 utility | 0.804 (0.274-0.997) | Beta | FIDELIO-DKD [13]  Versteegh et al. [23] |
| CKD4 utility | 0.793 (0.247-0.995) | Beta | FIDELIO-DKD [13]  Versteegh et al. [23] |
| CKD 5 w/o RRT utility | 0.772 (0.218-0.983) | Beta | FIDELIO-DKD [13]  Versteegh et al. [23] |
| Dialysis (acute) utility | 0.463 (0.000-0.732) | Beta | FIDELIO-DKD [13]  Versteegh et al. [23] |
| Dialysis (post-acute) utility | 0.463 (0.000-0.732) | Beta | FIDELIO-DKD [13]  Versteegh et al. [23] |
| Kidney Transplant (acute) utility | 0.656 (0.000-1.000) | Beta | FIDELIO-DKD [13]  Versteegh et al. [23] |
| Kidney Transplant (post-acute) utility | 0.722 (0.326-1.000) | Beta | FIDELIO-DKD [13]  Versteegh et al. [23] |
| Utility decrement associated with first MI (acute) | -0.039 (-0.018- -0.068) | Beta | FIDELIO-DKD [13]  Versteegh et al. [23] |
| Utility decrement associated with first MI (post-acute) | -0.039 (-0.018- -0.063) | Beta | FIDELIO-DKD [13]  Versteegh et al. [23] |
| Utility decrement associated with first stroke (acute) | -0.054 (-0.032- -0.068) | Beta | FIDELIO-DKD [13]  Versteegh et al. [23] |
| Utility decrement associated with first stroke (post-acute) | -0.054 (-0.032- -0.081) | Beta | FIDELIO-DKD [13]  Versteegh et al. [23] |
| Utility decrement associated with first hospitalization for HF (acute) | -0.042 (-0.025 – 0.063) | Beta | FIDELIO-DKD [13]  Versteegh et al. [23] |
| Utility decrement associated with first hospitalization for HF (post-acute) | -0.042 (-0.025 – 0.063) | Beta | FIDELIO-DKD [13]  Versteegh et al. [23] |
| Utility decrement associated with hyperkalaemia leading to hospitalisation | -0.005 (0.000- -0.025) | Beta | FIDELIO-DKD [13]  Versteegh et al. [23] |
| Utility decrement associated with new onset of atrial fibrillation / atrial flutter | 0.0000 | Beta | FIDELIO-DKD [13]  Versteegh et al. [23] |
| Utility decrement associated with hyperkalaemia not-leading to hospitalisation | -0.005 (0.000- -0.025) | Beta | FIDELIO-DKD [13]  Versteegh et al. [23] |
| Abbreviations: : ACE: Angiotensin-converting enzyme; ARB: Angiotensin receptor blockers CI: Confidence interval; CKD: Chronic kidney disease; CV: Cardiovascular; DDD: defined daily dose; DDP-4: Dipeptidyl Peptidase-4; eGFR: estimated glomerular filtration rate; GLP-1: glucagon-like peptide 1GP: General practitioner; HF: Heart failure; MI: Myocardial infarction; ; IS: Ischemic; ICH: intracerebral brain haemorrhage; RRT: Renal replacement therapy; SGLT2: Sodium-glucose Cotransporter-2 | | | |
