# Supplementary material for: Cost-effectiveness of finerenone in chronic kidney disease associated with type 2 diabetes in The Netherlands
Source: Cardiovasc Diabetol. 2023 Nov 28;22:328. doi: 10.1186/s12933-023-02053-6 (PMC10685667; doi:10.1186/s12933-023-02053-6)
Supplement: Supplementary file 10 — Additional file 10: Overview of assumptions that were used in the model. [file 12933_2023_2053_MOESM10_ESM.docx]

**Additional file 9**

**Table 1.** Overview of assumptions used in the model

| **Assumptions** |
| --- |
| Patients may experience up to one Main CV Event within a four-month cycle length; though rare, it is possible to experience more than one Main CV Event in real life. |
| OHEs do not affect the subsequent risk of CV events, CKD progression, or survival. |
| No treatment interruption; this overestimates treatment costs. |
| Constant efficacy of treatment; there is no evidence that the proportional hazard assumption was not met, so modelling of a time-varying HR is not performed |
| Memoryless assumption of Markov models; partly relaxed with composite health states tracking patients’ history |
| The model uses a cohort-level approach; therefore, subgroup analyses were performed. |
| It was assumed that patients stop finerenone after the median treatment duration in the FIDELIO analysis by applying a risk of 0.03 per patient per cycle to discontinue treatment, which both impacts costs and effects. Lifetime treatment duration was explored in a scenario. After RRT initiation all patients discontinue finerenone treatment. |
| It was assumed that in general patients do not stop SoC treatment. After RRT initiation 25% of patients discontinue treatment. |
| HRs were applied independently of significance level; ISPOR recommends that all known data should be incorporated for key parameters, including those that fall short of the conventional thresholds of statistical significance (although the group does not, however, define what they mean by ‘key’ parameters) [68]. |
| Similar utility values were used for health states / OHEs in all treatment arms; no evidence to suggest that treatment choice has any impact on QoL. |
| Medication groups used in the SoC were represented by an average of the three most described drugs that were recommended for CKD. Costs were based on the average costs per DDD. |
| Travel costs are not considered as these are already considered in the health state costs. |
| For the health state costs, a bottom-up approach was used that was validated by a clinical expert. |
| The number of kidney transplantations was low. Based on expert opinions, conducting a kidney transplant is dependent on donor availability rather than the treatments considered in the model. Experts also highlighted that patients with T2D are often ineligible for transplantation due to their numerous comorbidities. Therefore, the data found in the literature was an overestimation and the FIDELIO data was used. |
| Abbreviations: CKD: Chronic kidney disease DDD: Defined daily dose; ISPOR: Professional Society for Health Economics and Outcomes Research OHE: Other health event; SoC: Standard of care; T2D: Type 2 diabetes mellitus; |
